# The Impact of Vascular Risk Factors on Post-stroke Cognitive Impairment: The Nor-COAST Study

**DOI:** 10.3389/fneur.2021.678794

**Published:** 2021-08-05

**Authors:** Stina Aam, Mari Nordbø Gynnild, Ragnhild Munthe-Kaas, Ingvild Saltvedt, Stian Lydersen, Anne-Brita Knapskog, Hege Ihle-Hansen, Hanne Ellekjær, Rannveig Sakshaug Eldholm, Brynjar Fure

**Affiliations:** ^1^Department of Neuromedicine and Movement Science, Faculty of Medicine and Health Science, NTNU-Norwegian University of Science and Technology, Trondheim, Norway; ^2^Department of Geriatric Medicine, Clinic of Medicine, St. Olavs Hospital, Trondheim University Hospital, Trondheim, Norway; ^3^Stroke Unit, Clinic of Medicine, St. Olavs Hospital, Trondheim University Hospital, Trondheim, Norway; ^4^Department of Medicine, Vestre Viken Hospital Trust, Bærum Hospital, Drammen, Norway; ^5^Institute of Clinical Medicine, University of Oslo, Oslo, Norway; ^6^Department of Mental Health, Faculty of Medicine and Health Science, NTNU-Norwegian University of Science and Technology, Trondheim, Norway; ^7^Department of Geriatric Medicine, Oslo University Hospital, Oslo, Norway; ^8^Department of Internal Medicine, Central Hospital, Karlstad, Sweden; ^9^School of Medical Sciences, Örebro University, Örebro, Sweden

**Keywords:** post-stroke cognitive impairment, vascular dementia, stroke, vascular risk factors, cognition

## Abstract

**Introduction:** Post-stroke cognitive impairment (PSCI) is common, but evidence on the impact of vascular risk factors is lacking. We explored the association between pre-stroke vascular risk factors and PSCI and studied the course of PSCI.

**Materials and Methods:** Vascular risk factors were collected at baseline in stroke survivors (*n* = 635). Cognitive assessments of attention, executive function, memory, language, and the Montreal Cognitive Assessment (MoCA) were performed at 3 and/or 18 months post-stroke. Stroke severity was assessed with the National Institutes of Health Stroke Scale (NIHSS). PSCI was measured with global z; MoCA z-score; and z-score of the four assessed cognitive domains. Mixed-effect linear regression was applied with global z, MoCA z-score, and z-scores of the cognitive domains as dependent variables. Independent variables were the vascular risk factors (hypertension, hypercholesterolemia, smoking, diabetes mellitus, atrial fibrillation, coronary heart disease, previous stroke), time, and the interaction between these. The analyses were adjusted for age, education, and sex. There were between 5 and 25% missing data for the variables for PSCI.

**Results:** Mean age was 71.6 years (SD 11.7); 42% were females; and the mean NIHSS score at admittance was 3.8 (SD 4.8). Regardless of vascular risk factors, global z, MoCA, and all the assessed cognitive domains were impaired at 3 and 18 months, with MoCA being the most severely impaired. Atrial fibrillation (AF) was associated with poorer language at 18 months and coronary heart disease (CHD) with poorer MoCA at 18 months (LR = 12.80, *p* = 0.002, and LR = 8.32, *p* = 0.004, respectively). Previous stroke was associated with poorer global z and attention at 3 and 18 months (LR = 15.46, *p* < 0.001, and LR = 16.20, *p* < 0.001). In patients without AF, attention improved from 3 to 18 months, and in patients without CHD, executive function improved from 3 to 18 months (LR = 10.42, *p* < 0.001, and LR = 9.33, *p* = 0.009, respectively).

**Discussion:** Our findings indicate that a focal stroke lesion might be related to pathophysiological processes leading to global cognitive impairment. The poorer prognosis of PSCI in patients with vascular risk factors emphasizes the need for further research on complex vascular risk factor interventions to prevent PSCI.

## Introduction

Post-stroke cognitive impairment (PSCI) is prevalent and reported to be 53.4% in a recent review and meta-analysis of hospital-based studies (1). Recently published results from the STROKOG consortium showed global impairment in 44% of patients a short time after a stroke, with 30–35% of impairments in the following individual domains: attention and processing speed, memory, language, perceptual-motor function, and frontal executive function (2).

Knowledge about vascular risk factors as predictors of PSCI and its trajectories in patients with vascular risk factors is important because it might offer insights into the mechanisms of the disease and can be useful for the cognitive prognosis, and studies have shown contradicting results (3). Hypertension is a known risk factor for dementia; however, knowledge about its association with PSCI is scarce (2–5). Mid-life hypertension and smoking are associated with cognitive decline, while late-life hypertension alone might not be associated and may even be protective against dementia (3, 4, 6, 7). The STROKOG consortium found associations between cognition and diabetes mellitus, previous stroke, hypertension, atrial fibrillation, and smoking early after a stroke (2). Another recent study showed an association between cognition and blood pressure levels early after a stroke; however, these findings were explained by sociodemographic and clinical factors (8).

In a systematic review and meta-analysis of studies with both short- and long-term follow-ups after stroke, diabetes mellitus, atrial fibrillation, and previous stroke were shown to be predictors of post-stroke dementia (9). In the Oxford Vascular Study, post-stroke dementia was associated with previous stroke and diabetes mellitus in the long term following a stroke (10).

The aim of this study was to explore the association between pre-stroke vascular risk factors and cognitive impairment at 3 and 18 months post-stroke within both global cognitive measures and different cognitive domains. We also aimed to study the course of PSCI in patients with and without pre-stroke vascular risk factors.

## Methods

The study is part of the Norwegian Cognitive Impairment After Stroke (Nor-COAST) study, a multicenter prospective cohort study that recruited patients in five Norwegian stroke units from May 2015 through March 2017 (11–13). Inclusion criteria were hospitalization with acute ischemic or hemorrhagic stroke within 1 week after symptom presentation, fluency in a Scandinavian language, and age >18 years. The exclusion criterion was an expected survival of <3 months. The patients gave informed written consent for participation, and when a person was unable to do so, informed written consent was provided by his or her next of kin. The study was approved by the Regional Committee for Medical and Health Research Ethics (REC Nord 2015/171) and registered in ClinicalTrials.gov (NCT02650531). Further details are described in the previously published protocol article for the Nor-COAST study (11).

### Clinical Assessments

Demographic characteristics and vascular risk factors were collected from the patients' medical records. Hypertension was defined as pre-stroke use of antihypertensive medication or use of antihypertensive medication at discharge, hypercholesterolemia as pre-stroke use of lipid-lowering medication, smoking as current smoking, and diabetes mellitus as a history of diabetes mellitus noted in the medical records and/or pre-stroke use of antidiabetic medication and/or HbA1c ≥ 48 mmol/mol at admittance for stroke and/or use of antidiabetic medication at discharge. Atrial fibrillation included a history of permanent or paroxysmal atrial fibrillation or atrial flutter detected by electrocardiogram and described in the medical records and/or detected by electrocardiogram and/or telemetry during the hospital stay. Coronary heart disease was defined as a history of coronary heart disease according to the medical records, and previous stroke was defined as a history of previous stroke based on the medical records (12, 13). Stroke severity was assessed with the National Institutes of Health Stroke Scale (NIHSS) at admission (14). Etiology of ischemic strokes was classified according to the Trial of Org 10172 in Acute Stroke Treatment (TOAST) classification (15). TOAST modification was performed where the undetermined etiology of TOAST probable (15) first was classified as TOAST possible (15), then as TOAST likely (16) where patients with findings of carotid stenosis <50% were classified as having large artery disease (13).

### Cognitive and Functional Assessments

Cognitive function at 3- and 18-month follow-ups was assessed by a trained study staff using a cognitive test battery based on the National Institute of Neurological Disorders and Stroke–Canadian Stroke Network (NINDS–CSN) Harmonization Standards (17) adapted to validated cognitive tests in Norwegian (12, 13). The test battery comprised the Trail Making Tests Part A (TMT-A) and Part B (TMT-B) (time to completion) (18), Word List Memory and Recall Test and Verbal Fluency Test Category (animals) from the Consortium to Establish a Registry for Alzheimer's Disease (CERAD) battery (19, 20), the Verbal Fluency Test Letter (FAS) (21, 22), and the Montreal Cognitive Assessment (MoCA) (23), version 7.3 at the 3-month follow-up and version 7.1 at the 18-month follow-up. To minimize practice effect, the letter F in Verbal Fluency Test Letter (FAS) was retrieved from the MoCA. In addition, cognitive function was assessed with the Global Deterioration Scale (GDS) (24). Activities of daily living (ADL) were assessed with the Barthel Index (BI) (25) and global functional outcome with the Modified Rankin Scale (mRS) (26). GDS, ADL, and BI were performed at baseline and at 3- and 18-month follow-ups. Baseline assessments were performed during the hospital stay; 3- and 18-month follow-ups were performed at the hospitals' outpatient clinics. For patients unable to attend follow-up assessments, telephone interviews with the patients, their caregivers, or nursing home staff were conducted for assessment using the mRS, BI, GDS, and the Telephone MoCA (T-MoCA) (27).

### Cognitive Outcomes

Cognitive outcome assessments of the four domains included complex attention measured by the TMT-A, executive function by the TMT-B and FAS, memory by the Word List Delayed Recall, and language by the Verbal Fluency Test Category (12, 13, 28–31). Global cognition was also measured using the MoCA.

### Statistics

Z-scores normalized by mean and standard deviation (SD) of the normative data were derived from the raw scores of the cognitive tests, as described in Supplementary Table 1 (12, 13). PSCI was measured by global z, MoCA z-score, and z-scores of the four cognitive domains assessed. Global z was defined as the average of the four cognitive domains, which were measured by the z-score of the single completed cognitive test, except for executive function, measured by two tests where the average z-score was used. The z-scores were implemented with lower z-scores indicating poorer outcomes.

PSCI was analyzed with mixed-effect linear regression with global z, MoCA, and z-scores of four cognitive domains—attention, executive function, memory, and language—as dependent variables one at a time. The independent variables were the vascular risk factors (hypertension, hypercholesterolemia, smoking, diabetes mellitus, atrial fibrillation, coronary heart disease, previous stroke) examined one at a time, follow-up time, and the interaction between the vascular risk factor and follow-up time (model 1). We adjusted for age, education, and sex. The results for model 1 were presented as the estimates with mean and 95% confidence intervals (CI). In order to perform a hypothesis test for the effect of each vascular risk factor and follow-up time in model 1, the analyses were also performed with follow-up time (model 2) as well as with the vascular risk factor (model 3) as the independent variable. Hypothesis tests for the effects of vascular risk factors and follow-up times in model 1 were conducted by likelihood ratio tests comparing model 1 and model 2, as well as comparing model 1 and model 3. These results were presented as the test statistics with degrees of freedom and *p*-value.

In mixed-effect regression models, participants without available data at all time points are included in the analysis with data from available time points. Mixed-effect linear regression gives unbiased results if data are missing at random, while a complete case analysis would have been unbiased only under the stricter missing completely at random assumption (32). Data from clinical studies on cognition are often, to some degree, missing not at random, as those with poorer cognition are more likely to have missing data (33, 34). However, even if data are missing not at random, a method that is unbiased under a missing at random assumption results in less bias than a method assuming missing completely at random (34). There were between 5 and 25% missing data for the variables for PSCI. Imputation of outcome measures was performed as described in the Supplementary Material.

Sensitivity analyses with the exclusion of patients deceased at 18 months, as well as with exclusion of pre-stroke dementia defined as pre-stroke GDS 4–7, were performed to explore whether this affected the outcome. To assess the robustness of the results, we also performed unadjusted analyses and analyses adjusted for age, education, sex, pre-stroke mRS, and NIHSS altogether. An illustration of the statistical model for the mixed-effects linear regressions for model 1 is presented in Supplementary Figure 1.

Vascular risk factors, follow-up time, and sex were analyzed as categorical variables, while global z, MoCA z-score, and z-scores of the cognitive domains, age, education, mRS, and NIHSS were analyzed as continuous variables. Complete case analyses were used for vascular risk factors, age, education, and sex, while available case analyses were used for global z, MoCA, and z-scores of the cognitive domains, pre-stroke mRS, and NIHSS. Confounders were included as fixed effects, while subject and hospital were included as random effects.

Due to multiple hypotheses, we considered two-tailed *p*-values < 0.01 as statistically significant. Data were analyzed using SPSS 25 and STATA 16.0.

## Results

### Baseline Characteristics

**Figure 1:**
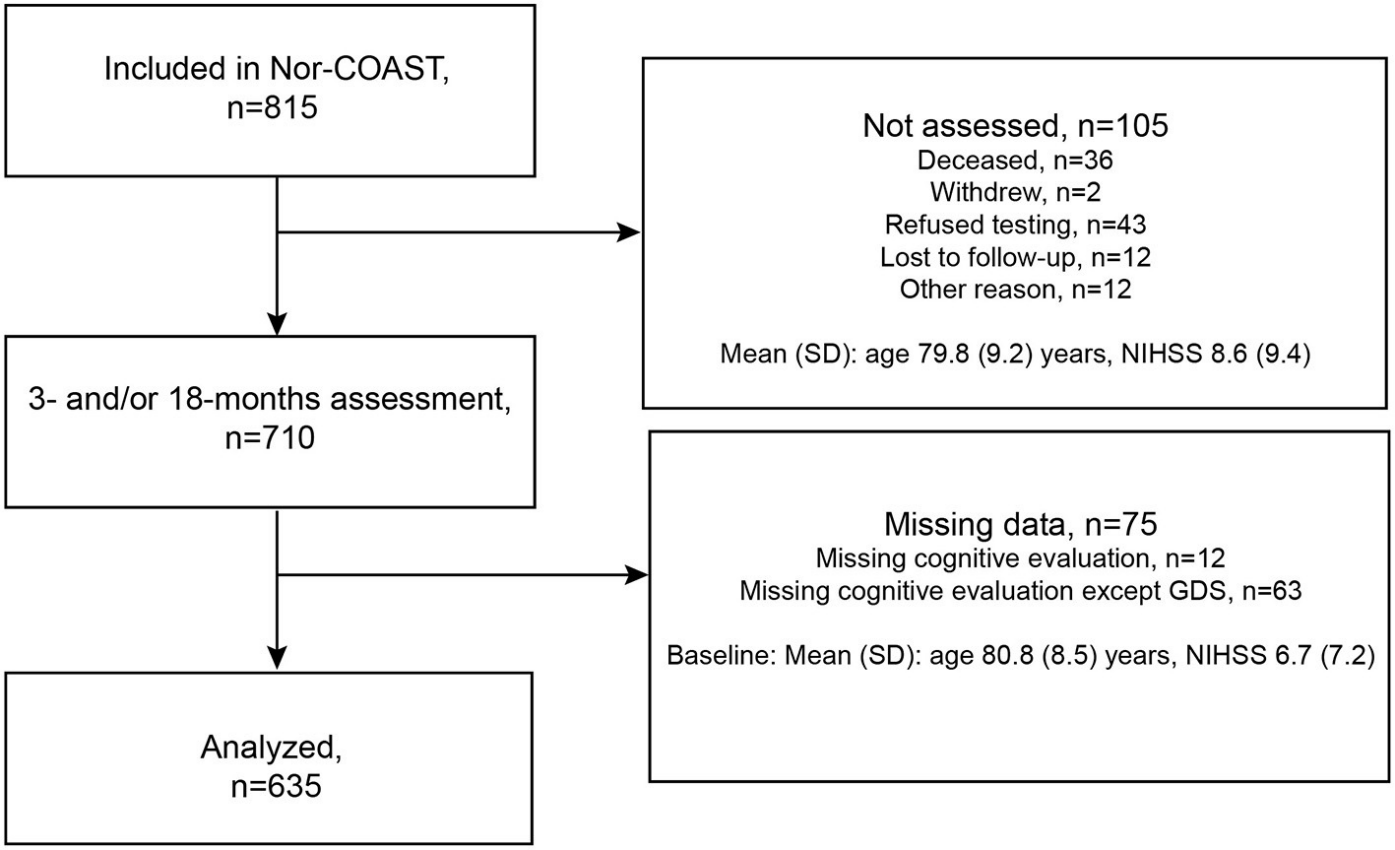
Flowchart of patients included in the study.

Of the 815 patients enrolled in the Nor-COAST study, 700 were assessed at the 3-month follow-up and 599 at the 18-month follow-up. Of the 599 patients assessed at the 18-month follow-up, 10 were not assessed at the 3-month follow-up. Of the 710 patients assessed at either 3 or 18 months, 75 were excluded due to missing cognitive data, and this resulted in a study sample of 635 patients (Figure 1). Of the 635 patients enrolled in the study, 21 were deceased at 18 months.

The mean age of the patients was 71.6 years (SD 11.7); 42% were females; the mean for years of education was 12.4 years (SD 3.8); and mean NIHSS score at admittance was 3.8 (SD 4.8). The baseline characteristics of the patients are shown in Table 1. Excluded patients had a mean age of 80.2 years (SD 9.0), 55% were females, mean education was 10.3 years (SD 3.0), and mean NIHSS score at admittance was 7.7 (8.5). The 21 patients assessed at 3 months and deceased at 18 months had a mean age of 74.2 years (SD 11.6), 29% were females, mean education was 12.2 years (SD 3.8), and mean NIHSS score at admittance was 4.5 (5.1). The numbers of patients completing cognitive tests for the cognitive domains, with mean z-score of the tests and proportions with z-score < −1.5, are shown in Table 2. Of the 605 and 508 patients assessed with MoCA at 3- and 18-month follow-ups, 21 and 25 patients, respectively, performed the telephone-MoCA.

**Table 1:** Baseline characteristics.

Demographics		*N* = 635		
	Mean age, years (SD)		71.6	(11.7)
	Female sex, *n* (%)		266	(42)
	Mean education, years (SD)		12.4	(3.8)
Vascular risk factors, *n* (%)				
	Hypertension, *n* (%)	*N* = 635	460	(72)
	Hypercholesterolemia, *n* (%)	*N* = 635	216	(34)
	Smoking, n (%)	*N* = 631	121	(19)
	Diabetes mellitus, *n* (%)	*N* = 635	145	(18)
	Mean BMI, kg/m^2^ (SD)	*N* = 600	26.1	(4.2)
	Atrial fibrillation, *n* (%)	*N* = 635	145	(23)
	Coronary heart disease, *n* (%)	*N* = 635	112	(18)
	Previous stroke, *n* (%)	*N* = 635	112	(18)
Stroke subtype, *n* (%)		*N* = 635		
	Cerebral infarction		582	(92)
	Cerebral hemorrhage		53	(8.3)
TOAST classification^*^, *n* (%)		*N* = 564		
	Large-vessel disease		140	(25)
	Cardioembolic disease		153	(27)
	Small-vessel disease		135	(24)
	Other etiology		17	(3.0)
	Undetermined etiology		119	(21)
Thrombolysis, *n* (%)		*N* = 629	153	(24)
Thrombectomy, *n* (%)		*N* = 635	12	(1.9)
Pre-stroke GDS (1–7), *n* (%)		*N* = 629		
	GDS = 1–2 (normal cognition)		568	(90)
	GDS = 3 (mild neurocognitive disorder)		36	(5.7)
	GDS = 4–7 (major neurocognitive disorder)		25	(4.0)
Assessments				
	NIHSS (0–42) at admittance, mean (SD)	*N* = 618	3.8	(4.8)
	Pre-stroke mRS (0–6), mean (SD)	*N* = 631	0.78	(1.0)
	mRS (0–6) at discharge, ^†^ mean (SD)	*N* = 633	2.1	(1.3)
	Barthel Index (0–100) at discharge, ^†^ mean (SD)	*N* = 633	89	(19)

*SD, standard deviation; BMI, body mass index; TIA, transient ischemic attack; TOAST, Trial of Org 10,172 in Acute Stroke Treatment; GDS, Global Deterioration Scale; NIHSS, National Institutes of Health Stroke Scale; mRS, modified Rankin Scale*.

* *TOAST modification (13); undetermined etiology of TOAST probable (15) was first classified as TOAST possible, (15) then as TOAST likely (16) where patients with findings of carotid stenosis <50% were classified as large artery disease. Finally, TOAST modified was developed by merging TOAST probable, TOAST possible, and TOAST likely*.

† *At discharge or day 7 if length of stay extends beyond 7 days*.

**Table 2:** Patients' performance on the global measures and cognitive domains.

	**3 months**					**18 months**				
	***N***	**Mean z-score (SD)**		***n*** **with** **z < −1.5 (%)**		***N***	**Mean z-score (SD)**		***n*** **with** **z < −1.5 (%)**	
Global z	560	−0.64	(1.26)	102	(18)	452	−0.47	(1.10)	59	(13)
MoCA	605	−1.18	(2.06)	211	(35)	508	−0.96	(2.08)	159	(27)
Attention	565	−1.00	(2.89)	129	(23)	454	−0.56	(2.42)	70	(15)
Executive function	558	−0.69	(1.48)	127	(23)	450	−0.46	(1.38)	89	(20)
Memory	492	−0.86	(1.37)	151	(31)	365	−0.79	(1.30)	94	(26)
Language	480	−0.63	(1.22)	103	(22)	339	−0.38	(1.39)	67	(20)

*SD, standard deviation; MoCA, Montreal Cognitive Assessment*.

### Impairments in Global Cognition and the Cognitive Domains

**Figure 2:**
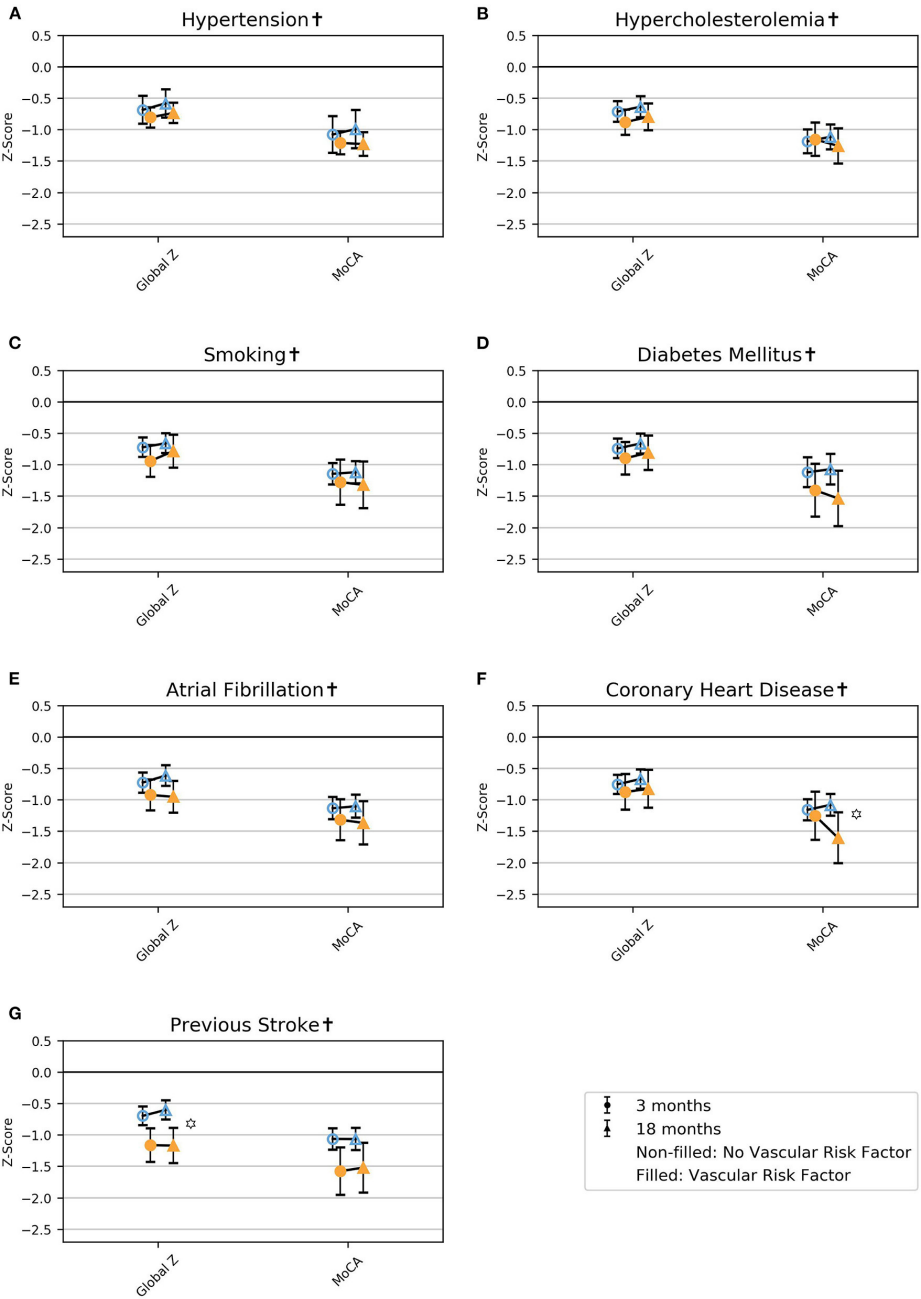
Mean z-score with 95% CI for the global cognitive measures for the different vascular risk factors at 3 and 18 months post-stroke in model 1. MoCA, Montreal Cognitive Assessment. **(A)** hypertension, **(B)** hypercholesterolemia, **(C)** smoking, **(D)** diabetes mellitus, **(E)** atrial fibrillation, **(F)** coronary heart disease, **(G)** previous stroke. ^†^Adjusted for age, education, and sex. 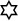
LRvasc χ^2^(2) *p* < 0.01.

**Figure 3:**
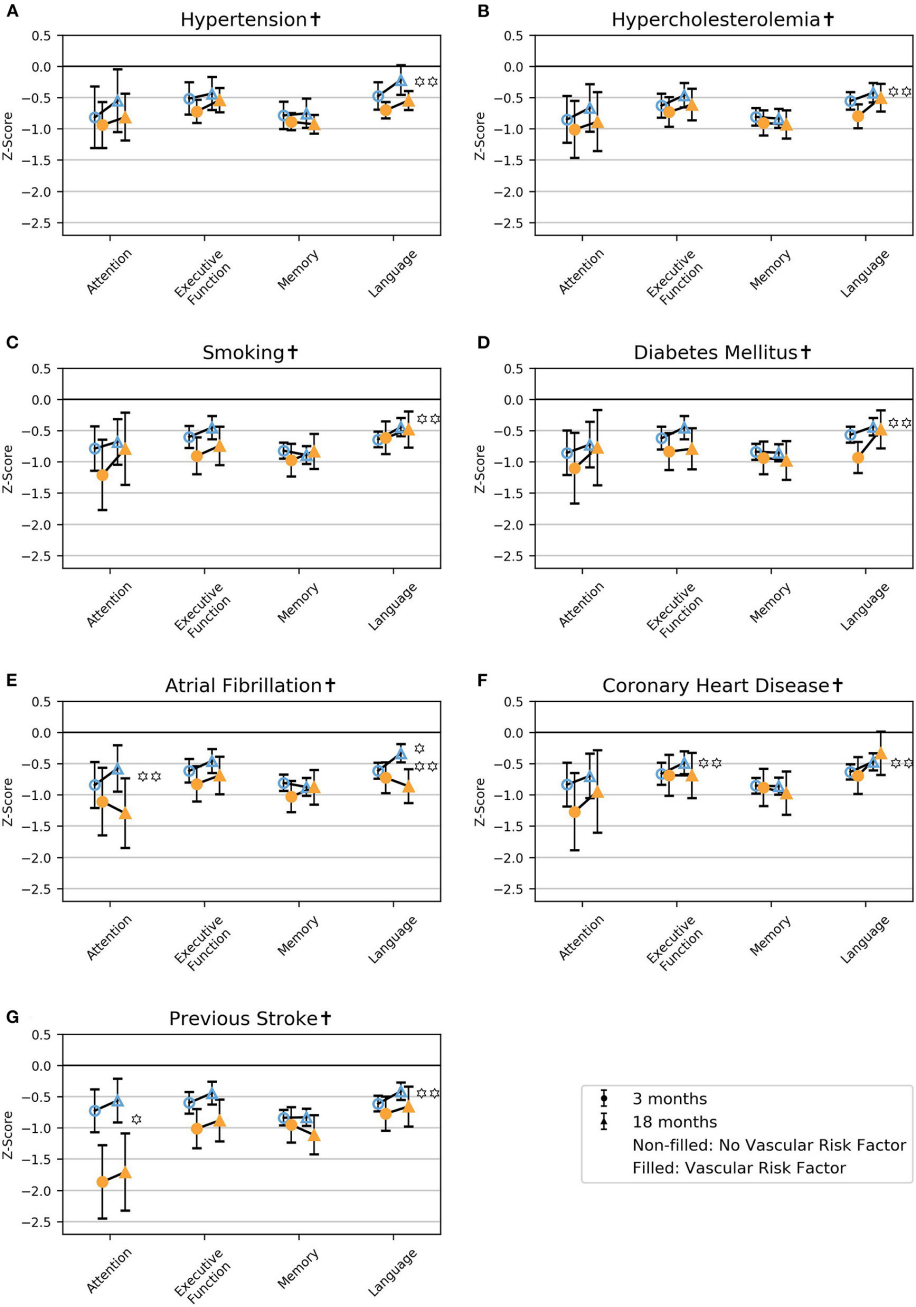
Mean z-score with 95% CI for the cognitive domains for the different vascular risk factors at 3 and 18 months post-stroke in model 1. **(A)** hypertension, **(B)** hypercholesterolemia, **(C)** smoking, **(D)** diabetes mellitus, **(E)** atrial fibrillation, **(F)** coronary heart disease, **(G)** previous stroke. ^†^Adjusted for age, education, and sex. 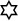
*p* < 0.01 for LRvasc χ^2^(2) = likelihood ratio test model 1 vs. model 2, with two degrees of freedom; hypothesis test of whether there is an effect of the vascular risk factor. 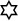

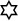
*p* < 0.01 LRtime χ^2^(2) = likelihood ratio test model 1 vs. model 2, with two degrees of freedom; hypothesis test of whether there is an effect of follow-up time.

Regardless of vascular risk factors, the global scores and the four cognitive domains (attention, executive function, memory, language) were impaired in terms of z-score < 0 at 3 and 18 months. In patients with vascular risk factors, MoCA and attention were the most severely impaired, while language was the least severely impaired. In contrast, patients without vascular risk factors showed a more equally distributed severity of impairments across global measures and cognitive domains (Figures 2, 3A–G; Supplementary Table 2).

### Differences in Cognitive Function

Atrial fibrillation was associated with poorer language at 18 months, and coronary heart disease was associated with poorer performance on the MoCA at 18 months (Figure 3E; Figure 2F; and Supplementary Table 2). Previous stroke was associated with poorer global z and attention at both 3 and 18 months (Figures 2G, 3G; Supplementary Table 2).

### Course of Cognition

In patients without atrial fibrillation, attention improved from 3 to 18 months, and in patients without coronary heart disease, executive function improved from 3 to 18 months (Figures 3E,F; Supplementary Table 2). Language improved from 3 to 18 months in patients with hypercholesterolemia, diabetes mellitus, or coronary heart disease and in nonsmokers and patients without hypertension, atrial fibrillation, or previous stroke (Figures 3A–G; Supplementary Table 2).

### Sensitivity Analyses

The results were essentially the same for sensitivity analyses; unadjusted analyses; analyses excluding patients deceased at 18 months (*n* = 21) adjusted for age, education, and sex; analyses excluding patients with pre-stroke dementia (*n* = 25) adjusted for age, education, and sex; and analyses adjusted for age, education, sex, pre-stroke mRS, and NIHSS altogether (Supplementary Figures 2–9; Supplementary Tables 3–6). The exceptions were that the improvement in attention for patients without atrial fibrillation did not reach statistical significance for exclusion of pre-stroke dementia; the improvement in executive function in patients without coronary heart disease did not reach statistical significance for analyses with the exclusion of deceased patients, exclusion of pre-stroke dementia, and analyses adjusted for age, education, sex, pre-stroke mRS, and NIHSS; the effect of previous stroke did not reach statistical significance for global z for analyses adjusted for age, education, sex, pre-stroke mRS, and NIHSS; the improvement in language in non-smokers did not reach statistical significance for analyses with the exclusion of deceased patients and analyses adjusted for age, education, sex, pre-stroke mRS, and NIHSS.

The numbers of patients with the different vascular risk factors included in the analyses are shown in Supplementary Table 7.

## Discussion

We identified impairments in the global measures and all the assessed cognitive domains regardless of pre-stroke vascular risk factors in this observational study of stroke survivors. Coronary heart disease and previous stroke were associated with poorer global cognition, previous stroke with poorer attention, and atrial fibrillation with poorer language. We found improvement in attention in patients without atrial fibrillation and in executive function in patients without coronary heart disease.

Our findings of poorer cognition in patients with atrial fibrillation, coronary heart disease, and previous stroke align with Lo et al.'s findings of associations between cognition and diabetes mellitus, previous stroke, hypertension, atrial fibrillation, and smoking (2). We were unable to measure exposure to pre-stroke vascular risk factors over time, and this could explain our lack of a finding for hypertension in our study population with its relatively high average age. It has been shown that mid-life hypertension and smoking are associated with cognitive decline, whereas late-life hypertension alone might not be associated (3, 4, 6, 7), maybe an effect of lower burden of hypertension over time, although selection bias cannot be ruled out as an explanation. Atrial fibrillation, coronary heart disease, and previous stroke can be seen as risk factors that have already exerted an influence on the functioning of the heart, brain, or other organs, indicating a long-lasting and severe exposure to vascular risk factors that may explain our findings.

Although a stroke lesion is focal, we found the most severe global cognitive impairment in patients with pre-stroke vascular risk factors, which might indicate that vascular risk factors contribute to decline in global, rather than in focal, cognitive function. The MoCA, followed by attention, was the most severely impaired regardless of vascular risk factors. The MoCA measures a broad spectrum of domains, broader than the global z in this study, and is a global assessment (23). Attention should probably be seen as an expression of global rather than focal cognition (35). Therefore, our results emphasize the global cognitive impairment seen after a stroke. However, there is also a possibility that impairment in a cognitive domain is a spillover effect from global cognitive impairment or impairment in other cognitive domains. The broader spectrum of the MoCA, in addition to the potential underestimation of between cognitive domain differences with imputation of global z, could be an explanation for the MoCA being more impaired and also for the different patterns when this was seen for these two global measures. Lacking a stroke-free control group, we were unable to evaluate whether cognition is more severely impaired in those who have suffered a stroke than in the background population. A recent study found no differences in cognitive function between patients with minor stroke and those with myocardial infarction 1 year after the vascular event (36). Additionally, in our study population comprising both first-ever and recurrent strokes, an evaluation of the effects of recurrent strokes is limited.

Memory was severely impaired regardless of vascular risk factors, with no progression over time, which may indicate a neurodegenerative component compatible with Alzheimer's disease (AD), especially for the oldest age groups, as AD is more strongly associated with memory impairment than vascular cognitive impairment is (3). As neurodegenerative processes typically develop slowly, we might have captured a decline in memory with a longer follow-up time. Although the results for both global cognition and cognitive domains remained almost the same when patients with pre-stroke dementia were excluded, we were unable to determine the impact of neurodegenerative components on PSCI. Vascular factors are also shown to be established risk factors for cognitive decline in Alzheimer's disease (37), and the global impairments seen in AD might be related to vascular risk factors.

Poorer language skills were identified in patients with atrial fibrillation, which is probably related to focal cortical lesions in the dominant hemisphere (2). Regardless of vascular risk factors, there was an improvement in language from 3 to 18 months, which aligns with the findings of Maas et al. of good prognoses in patients with post-stroke aphasia (38). The improvement we found is more likely related to the improvement in language in the entire stroke population we have shown in a previous work (13). Most patients in Norway with aphasia after suffering a stroke receive speech rehabilitation from a speech therapist according to the Norwegian guidelines for stroke treatment (39). However, we had no data on rehabilitation, and we were unable to conclude whether the improvement was due to natural brain regeneration or rehabilitation.

In previous publications, we have shown that about half of stroke survivors experience PSCI, and most have mild neurocognitive disorders (12, 13). We found improvement in attention and executive function in patients without vascular risk factors. Studies focusing on the prevention of PSCI and improvement in PSCI and studies designed to prevent deterioration of PSCI over time are critically important. Cochrane reviews have identified a lack of knowledge on the effects of cognitive rehabilitation for attention and executive function in stroke populations and call for more research to clarify the impact of cognitive rehabilitation on PSCI (40, 41). Our findings of improvements in a subgroup support the need for such research.

Primary and secondary prevention of stroke is shown to decrease the risk of dementia (42), and the poorer prognoses in patients with pre-stroke vascular risk factors we identified emphasize a need for a preventive vascular approach to keep these risk factors at a minimum. There is a lack of knowledge about which vascular risk factors are most important for the prognosis of PSCI, and intervention aimed at single vascular risk factors may not be effective in preventing PSCI. In both a general and a stroke population, the presence of several vascular risk factors is shown to be associated with a higher risk of dementia than only one or two such factors (43–45).

However, as previous randomized controlled studies with low power and short follow-up time (46, 47) have failed to show any effect on cognition after stroke, the role of multifactorial interventions in preventing PSCI is still unclear. A systematic review concluding that recurrent stroke rather than vascular risk factors is the explanation for incident dementia (44, 48) aligns with our findings of the most severe impairments in global cognitive measures and attention in patients with previous stroke. This emphasizes the critical need to prevent recurrent stroke in order to prevent cognitive impairment.

This study has several strengths. Its first is a large sample size and multicenter design with longitudinal cognitive assessments of most cognitive domains in both the early period and long term after a stroke. A second strength is a study population with similar baseline characteristics to a Norwegian stroke population, although patients with more severe strokes and older age were unable to complete the entire test battery and, thereby, less likely to contribute to this study's findings (49, 50). A third strength is standardization with z-scores and minimization of selection bias by using mixed-effect linear regression models.

The study also has several limitations. First, the lack of a control group results in a descriptive study not designed to study causality, where adjustment for several confounders could result in overadjustment (51). Second, we lack Norwegian normative data. Third, all the cognitive domains except executive function are measured by only one cognitive test, and a cognitive test for visuospatial function beyond this subdomain in MoCA is lacking. Fourth, the absence of neuroimaging data limited the possibility to evaluate the effect of regional brain damage from infarctions and long-term ischemia. Use of the TOAST classification could have provided some insight into this by differentiating between lacunar and territorial strokes. However, in a previous publication, we found no statistically significant differences in cognitive domains across stroke subtypes, except from attention (13). Due to the risk of overadjustment, the TOAST classification was not included in the analyses of the present study. Fifth, the inclusion of patients in the acute phase of stroke when most of the population has temporarily elevated blood pressure limited the definition of hypertension to “use of antihypertensive medication,” and this might introduce a misclassification bias. To minimize bias from the participants with undetected pre-stroke hypertension, we defined hypertension as pre-stroke use of antihypertensive medication and/or use of antihypertensive medication at discharge. Hypertension defined as “use of antihypertensive medications” will also include users of antihypertensive medications for other reasons than hypertension, comprising mainly participants with coronary heart disease or heart failure and, thus, resulting in an overestimation of hypertension and potentially weakening an association with PSCI. Sixth, exploring the vascular risk factors one at a time in a cohort with a considerable level of at least one vascular risk factor complicates identification of the vascular contribution to cognition, and an alternative way could be measuring the burden of vascular risk factors by the number of these factors. Seventh, inclusion of both ischemic and hemorrhagic strokes sharing several common vascular risk factors, although not identical for the two etiologies (52), could weaken the associations. However, in a previous publication, we found no statistically significant differences in cognitive domains across ICH and the ischemic stroke subtypes. The results, therefore, should be interpreted with caution.

## Conclusion

Our findings of severely impaired global cognitive function indicate that a focal stroke lesion might be related to pathophysiological processes leading to global cognitive impairment. The poorer prognoses of PSCI in patients with vascular risk factors emphasize the need for further research focusing on the effectiveness of a complex intervention targeting all risk factors to prevent PSCI, preferably with a randomized controlled design.

## Data Availability Statement

The datasets generated for this article are not readily available because of Norwegian regulations and conditions for informed consent. Requests to access the datasets should be directed to Ingvild Saltvedt, ingvild.saltvedt@ntnu.no.

## Ethics Statement

The studies involving human participants were reviewed and approved by Regional Committee for Medical and Health Research Ethics (REC Nord 2015/171), UiT Norges arktiske universitiet, Postboks 6050 Langnes, 9037 Tromsø. The patients/participants provided their written informed consent to participate in this study.

## Author Contributions

IS manages the Nor-COAST study and developed the idea for the design of the present study. SA, IS, and BF were responsible for the analysis plan and writing the present report. SA and SL planned the statistical analyses and SA performed them. MG was responsible for the workup with categorization of the vascular risk factors. RM-K, HI-H, and HE were responsible for collecting data at their respective hospitals. A-BK and RE contributed to the analysis plan. All authors interpreted the results, read, and approved the final manuscript.

## Conflict of Interest

IS and A-BK have been investigators in the Boehringer Ingelheim drug trial 1346.0023, and A-BK has also been an investigator for Roche BN29553. The remaining authors declare that the research was conducted in the absence of any commercial or financial relationships that could be construed as a potential conflict of interest.

## Publisher's Note

All claims expressed in this article are solely those of the authors and do not necessarily represent those of their affiliated organizations, or those of the publisher, the editors and the reviewers. Any product that may be evaluated in this article, or claim that may be made by its manufacturer, is not guaranteed or endorsed by the publisher.
