# Supplementary material for: The Impact of Vascular Risk Factors on Post-stroke Cognitive Impairment: The Nor-COAST Study
Source: Front Neurol. 2021 Aug 5;12:678794. doi: 10.3389/fneur.2021.678794 (PMC8374055; doi:10.3389/fneur.2021.678794)
Supplement: Supplementary file 1 [file Data_Sheet_1.pdf]

## SUPPLEMENTARY MATERIAL

### Imputation of cognitive outcome measures

To minimize selection bias from excluded patients, imputation of cognitive outcome measures was performed as described in previous work in the Nor-COAST study and in the following (1, 2). Single items missing in the MoCA total scores were imputed by the mean of the available MoCA items for the same patient (n= 2 at 3 months follow-up and n=4 at 18 months follow-up). For patients assessed with telephone-MoCA (3) , 8 of 30 points of MoCA could not be assessed by telephone, and these 8 points were imputed by the mean of the available MoCA items for the same patient (n=21 at 3 months follow-up of whom 3 patients had one single item missing in MoCA in addition to the 8 points not assessed, and n=25 at 18 months follow-up of whom 6 patients had items missing in MoCA in addition to the 8 points not assessed). For the patients able to start but not completing the TMT-A (n=14 at 3 months follow-up, and n=8 at 18 months follow-up) and TMT-B (n=91 at 3 months follow-up, and n=57 at 18 months follow-up) due to cognitive impairment, the tests' results were set as equal to the time of the interruption of the tests, which was 300 seconds for both (1, 2, 4). For the global z, there was a trade-off between the detection of post-stroke domain differences and minimizing bias from missing data. However, as those with poorer cognition are more likely to have missing data (5, 6), we imputed missing values on the domains' z-scores using the mean z-scores from the other domains for the same patient at the same time point, if z-scores were available for at least 2 of 4 domains (n=129 at 3-month follow-up, and n=127 at 18-month follow-up) to minimize bias from missing data. Other missing data were not imputed but treated as missing.

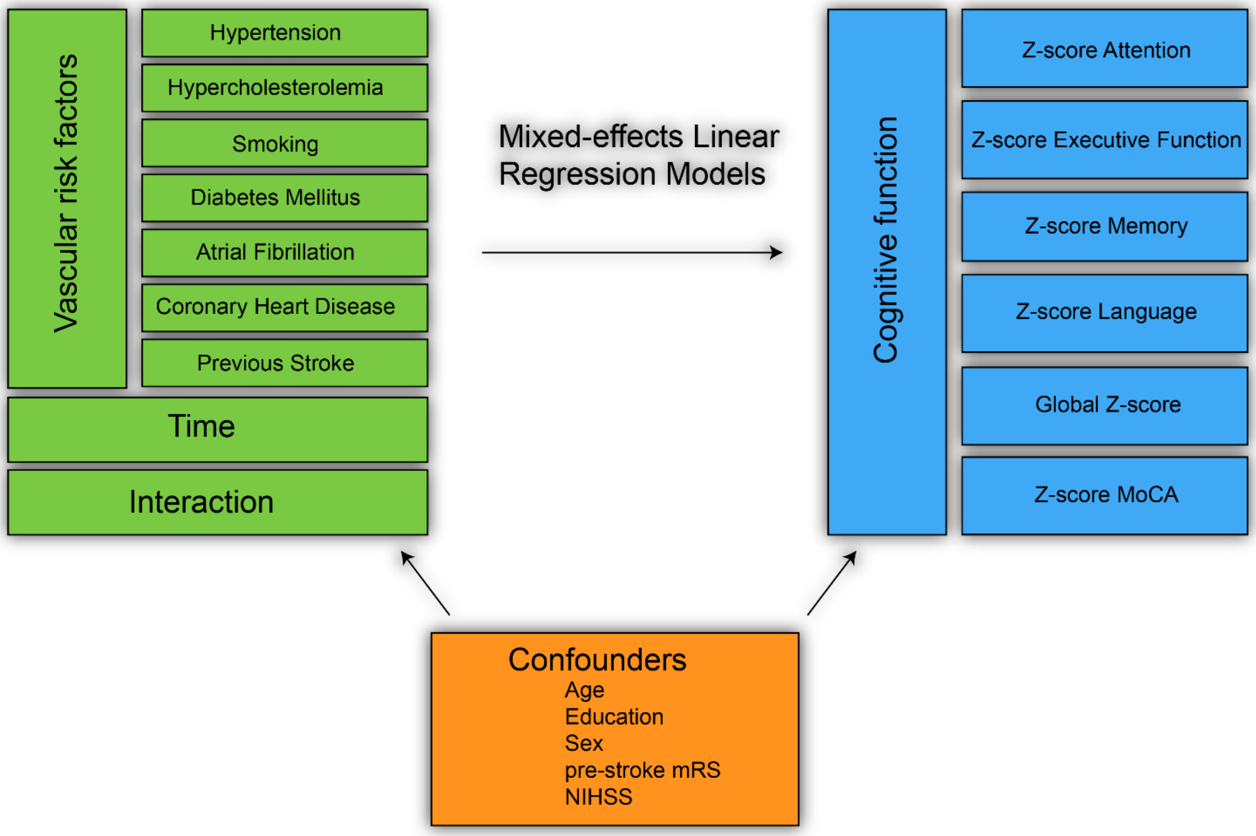


**Figure S1. Illustration of the mixed-effects linear regression for model 1.**

The outcome variables of cognitive function as well as the vascular risk factors were analyzed one at a time. Follow-up time and the interaction between the vascular risk factor and follow-up time were included in all analyses. The main analyses were adjusted for age, education, and sex. Also, unadjusted analyses were performed as well as analyses adjusted for age, education, sex, pre-stroke mRS, and NIHSS altogether.

| Supplementary Table S1. Normative data for the cognitive test battery | |
|---|---|
| **Cognitive Test** | **Normative data** |
| Trail Making Test A (TMT-A) and B (TMT-B) | For patients ages 18–59 years or >80 years:<br>Trail Making Test A and B: Normative data stratified by age and education (7)<br><br>For patients ages 60–79 years:<br>Age-, Sex-, and Education-Specific Norms for an Extended CERAD<br>Neuropsychological Assessment Battery–Results From the Population-Based LIFE-Adult-Study (8) |
| Word List Recall | For patients ages < 60 years:<br>Consortium to Establish a Registry for Alzheimer's Disease (CERAD). Part V. A normative study of the neuropsychological battery (9)<br><br>For patients ages 60–79 years:<br>Age-, Sex-, and Education-Specific Norms for an Extended CERAD<br>Neuropsychological Assessment Battery–Results From the Population-Based LIFE-Adult-Study (8)<br><br>For patients ages > 80 years:<br>CERAD-NP battery: Age-, gender- and education-specific reference values for selected subtests. Results of the German Study on Ageing, Cognition and Dementia in Primary Care Patients (AgeCoDe) (9) |
| Verbal Fluency Test Letters (FAS) | Normative data stratified by age and education for two measures of verbal fluency: FAS and animal naming (10) |
| Verbal Fluency Test Category (animals) | For patients aged 18–59 years or >80 years:<br>Normative data stratified by age and education for two measures of verbal fluency: FAS and animal naming (10)<br><br>For patients ages 60–79 years:<br>Age-, Sex-, and Education-Specific Norms for an Extended CERAD<br>Neuropsychological Assessment Battery–Results From the Population-Based LIFE-Adult-Study (8) |
| Montreal Cognitive Assessment (MoCA) | Montreal Cognitive Assessment: Normative data from a large Swedish population-based cohort (11) |

| Table S2. Hypothesis test of whether there is an effect of the vascular risk factor and follow-up time in model 1 for the global measures and cognitive domains for the different vascular risk factors for analyses adjusted for age, education and sex | | | | | | | | |
|---|---|---|---|---|---|---|---|---|
| | | **Hypertension** | **Hypercholesterolemia** | **Smoking** | **Diabetes mellitus** | **Atrial fibrillation** | **Coronary heart disease** | **Previous stroke** |
| **Global z** | $LR_{vasc}$ $\chi2(2)$, p-value | 1.59, 0.452 | 2.39, 0.302 | 3.09, 0.214 | 1.39, 0.498 | 6.99, 0.030 | 1.02, 0.601 | 12.76, 0.002* |
| | $LR_{time}$ $\chi2(2)$, p-value | 5.56, 0.062 | 5.30, 0.071 | 6.75, 0.034 | 5.30, 0.071 | 8.24, 0.016 | 5.46, 0.065 | 6.40, 0.041 |
| **MoCA** | $LR_{vasc}$ $\chi2(2)$, p-value | 1.71, 0.425 | 1.94, 0.379 | 0.90, 0.825 | 1.48, 0.478 | 1.75, 0.186 | 8.32, 0.004* | 6.08, 0.108 |
| | $LR_{time}$ $\chi2(2)$, p-value | 0.61, 0.738 | 1.90, 0.386 | 0.23, 0.891 | 1.12, 0.570 | 0.35, 0.840 | 6.61, 0.010 | 0.13, 0.936 |
| **Attention** | $LR_{vasc}$ $\chi2(2)$, p-value | 1.24, 0.537 | 0.80, 0.669 | 3.70, 0.158 | 1.29, 0.524 | 8.77, 0.013 | 2.12, 0.347 | 16.20, <0.001* |

| | | | | | | | | |
|---|---|---|---|---|---|---|---|---|
| | $LR_{time}$ $\chi2(2)$, p-value | 5.19, 0.075 | 4.63, 0.099 | 7.39, 0.025 | 5.38, 0.068 | 10.42, <0.01* | 5.11, 0.078 | 4.41, 0.110 |
| **Executive function** | $LR_{vasc}$ $\chi2(2)$, p-value | 2.37, 0.305 | 1.20, 0.550 | 4.30, 0.117 | 4.00, 0.135 | 2.50, 0.286 | 1.62, 0.445 | 8.06, 0.018 |
| | $LR_{time}$ $\chi2(2)$, p-value | 8.77, 0.013 | 8.13, 0.017 | 7.87, 0.020 | 8.62, 0.013 | 8.10, 0.017 | 9.33, 0.009* | 7.96, 0.019 |
| **Memory** | $LR_{vasc}$ $\chi2(2)$, p-value | 1.52, 0.677 | 2.18, 0.537 | 2.26, 0.521 | 0.63, 0.889 | 3.27, 0.352 | 0.36, 0.948 | 2.62, 0.454 |
| | $LR_{time}$ $\chi2(2)$, p-value | 0.42, 0.812 | 0.19, 0.908 | 2.23, 0.329 | 0.14, 0.931 | 2.31, 0.316 | 0.32, 0.854 | 1.14, 0.565 |
| **Language** | $LR_{vasc}$ $\chi2(2)$, p-value | 5.53, 0.063 | 4.38, 0.112 | 0.20, 0.904 | 7.88, 0.019 | 12.80, 0.002* | 1.26, 0.531 | 2.04, 0.361 |
| | $LR_{time}$ $\chi2(2)$, p-value | 10.27, 0.006* | 11.38, 0.003* | 9.67, 0.008* | 13.65, 0.001* | 18.58, <0.001* | 11.18, 0.004* | 10.05, 0.007* |

MoCA=Montreal Cognitive Assessment.
$LR_{vasc}$ $\chi2(2)$ =Likelihood ratio test model 1 vs model 2, with two degrees of freedom; hypothesis test of whether there is an effect of the vascular risk factor.
$LR_{time}$ $\chi2(2)$ =Likelihood ratio test model 1 vs model 3, with two degrees of freedom; hypothesis test of whether there is an effect of follow-up time.
*p<0.01

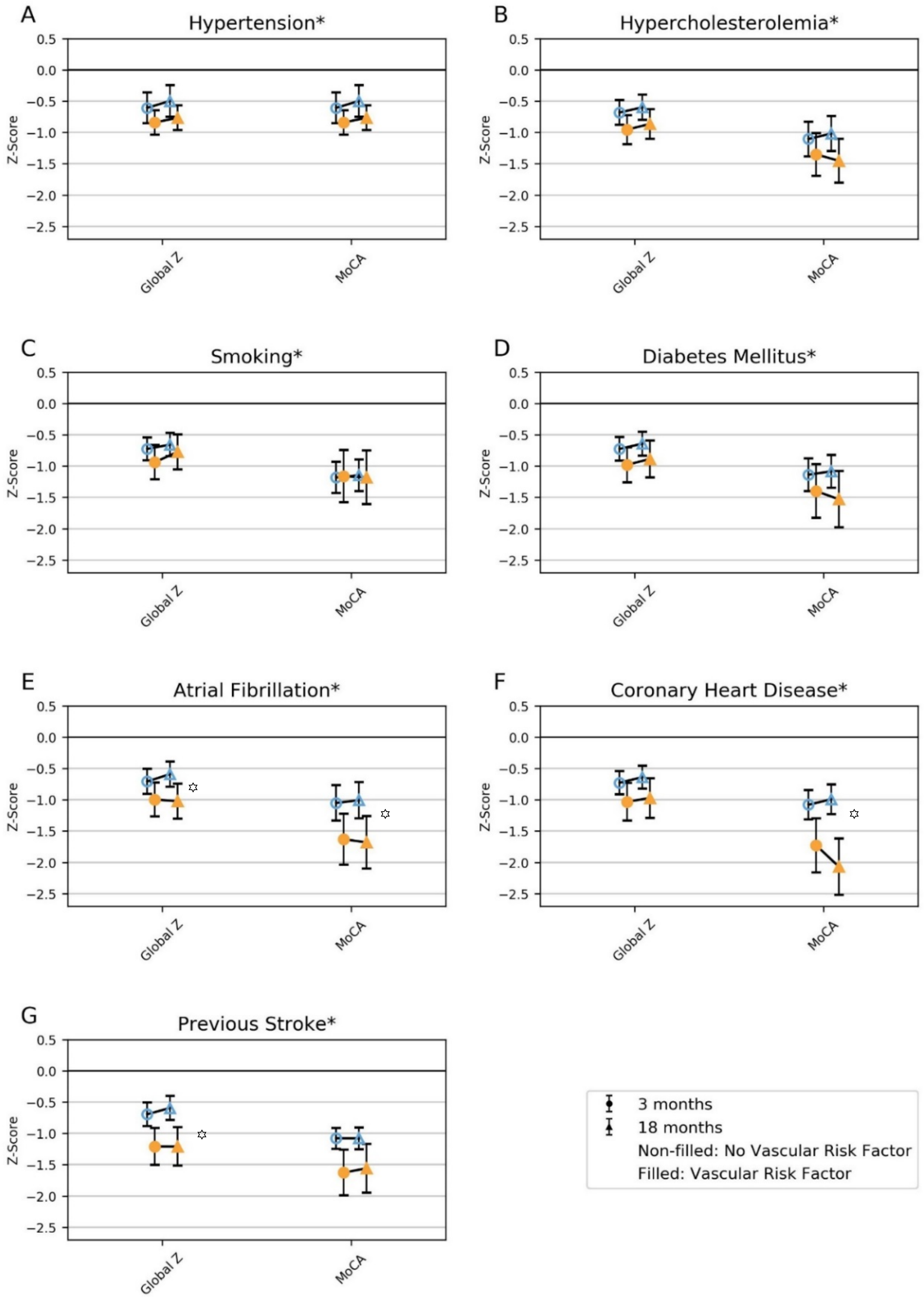


**Figure S2. Sensitivity analyses without adjustment: Mean z-scores with 95% confidence intervals for for the global cognitive measures for the different vascular risk factors at 3- and 18-months post-stroke in model 1**

MoCA = Montreal Cognitive Assessment

*unadjusted analysis

✡LRvasc $\chi 2(2)$ $p<0.01$, ✡✡LRtime $\chi 2(2)$ $p<0.01$

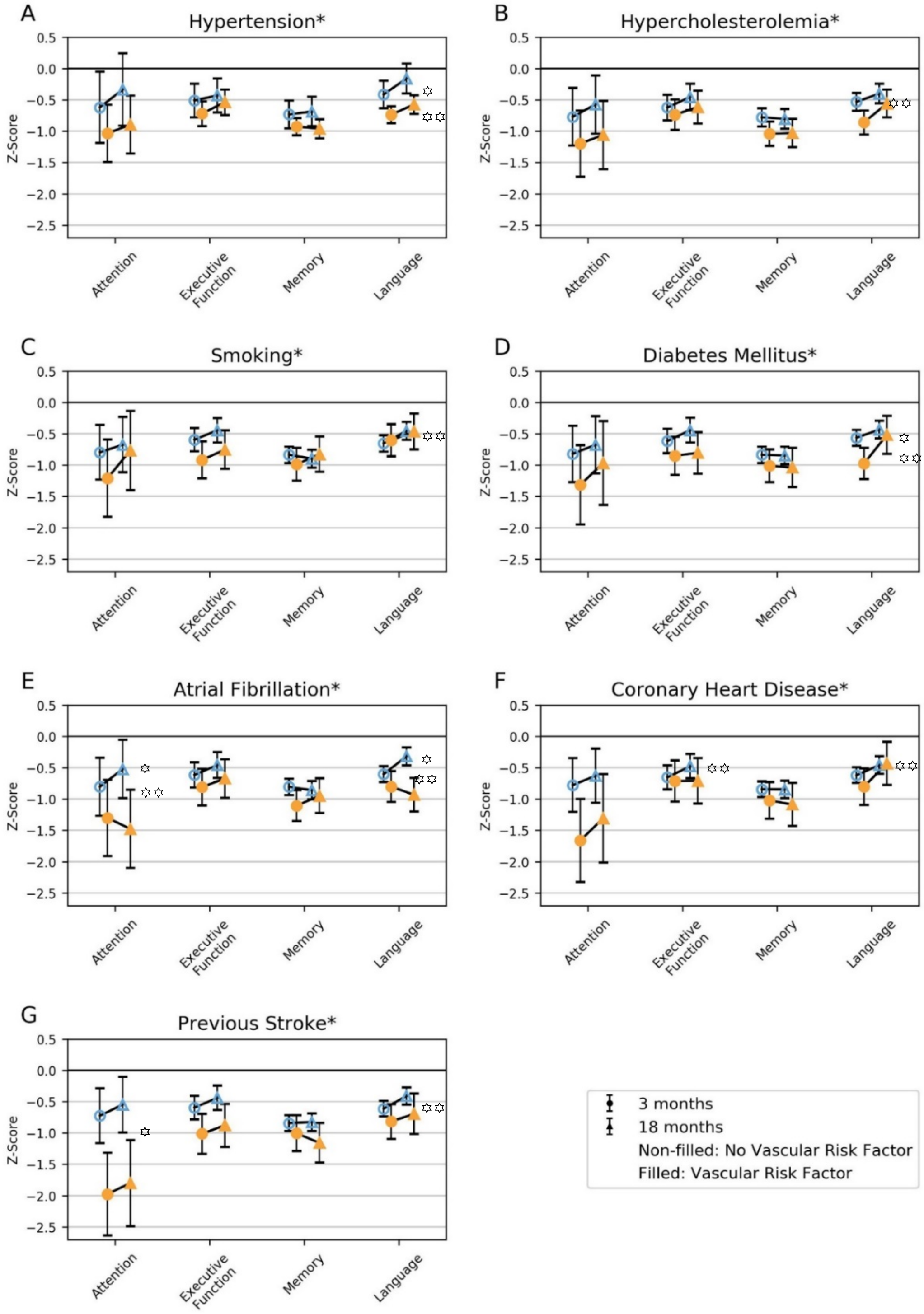


**Figure S3. Sensitivity analyses without adjustment: Mean z-score with 95% CI for the cognitive domains for the different vascular risk factors at 3- and 18-months post-stroke in model 1**

*unadjusted analysis

✡LRvasc $\chi 2(2)$ $p<0.01$, ✡✡LRtime $\chi 2(2)$ $p<0.01$

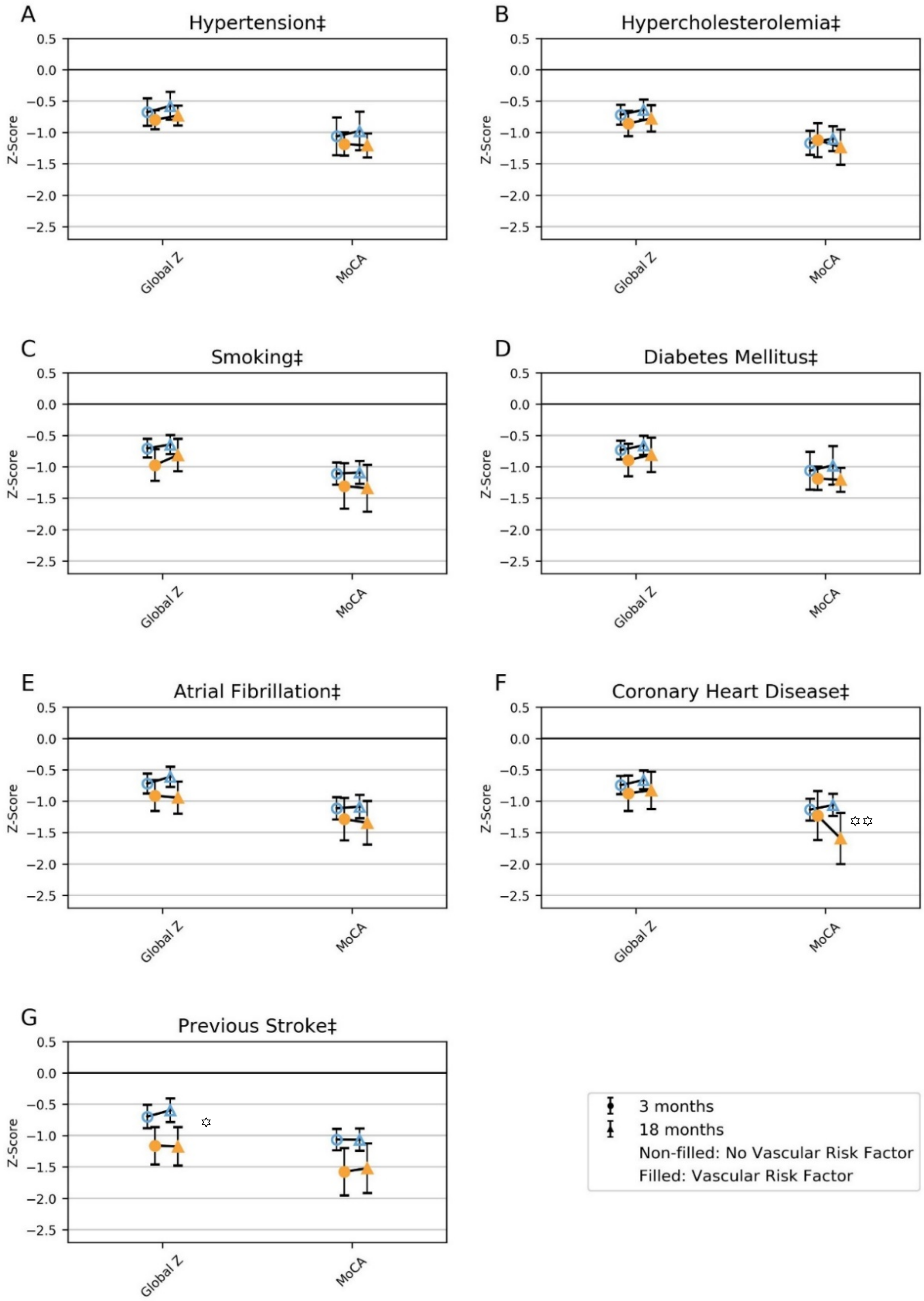


**Figure S4. Sensitivity analyses with exclusion of patients deceased at 18 months: Mean z-scores with 95% confidence intervals for the global cognitive measures for the different vascular risk factors at 3- and 18-months post-stroke, adjusted for age, education and sex in model 1**

MoCA = Montreal Cognitive Assessment

‡ exclusion of patients deceased at 18 months, adjusted for age, education, and sex

[✡]LRvasc $\chi 2(2)$ $p<0.01$, [✡✡]LRtime $\chi 2(2)$ $p<0.01$

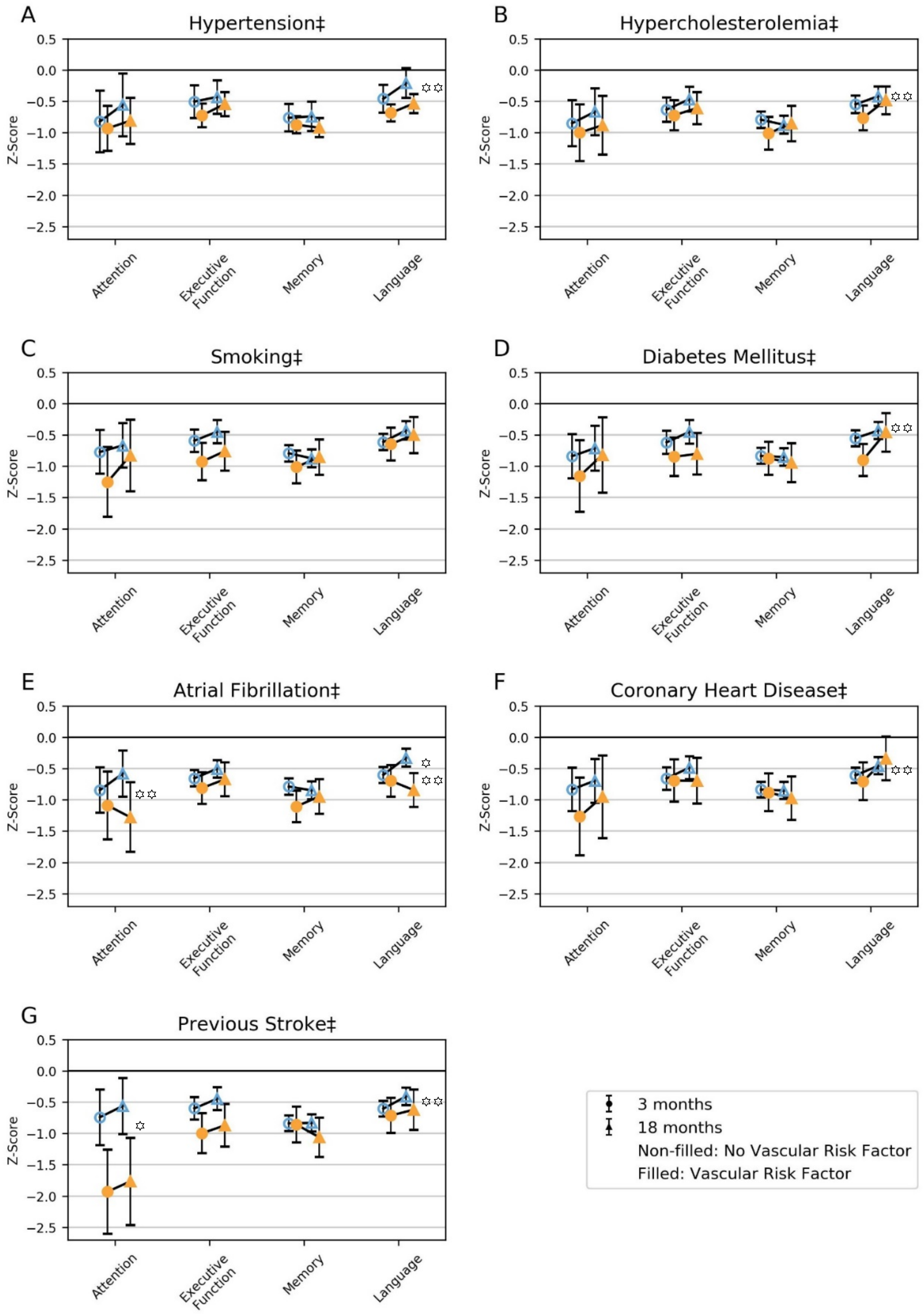


**Figure S5. Sensitivity analyses with exclusion of patients deceased at 18 months: Mean z-scores with 95% confidence intervals for the cognitive domains for the different vascular risk factors at 3- and 18-months post-stroke, adjusted for age, education and sex in model 1**

‡ exclusion of patients deceased at 18 months, adjusted for age, education, and sex

☼LRvasc $\chi2(2)$ $p<0.01$, ☼☼LRtime $\chi2(2)$ $p<0.01$

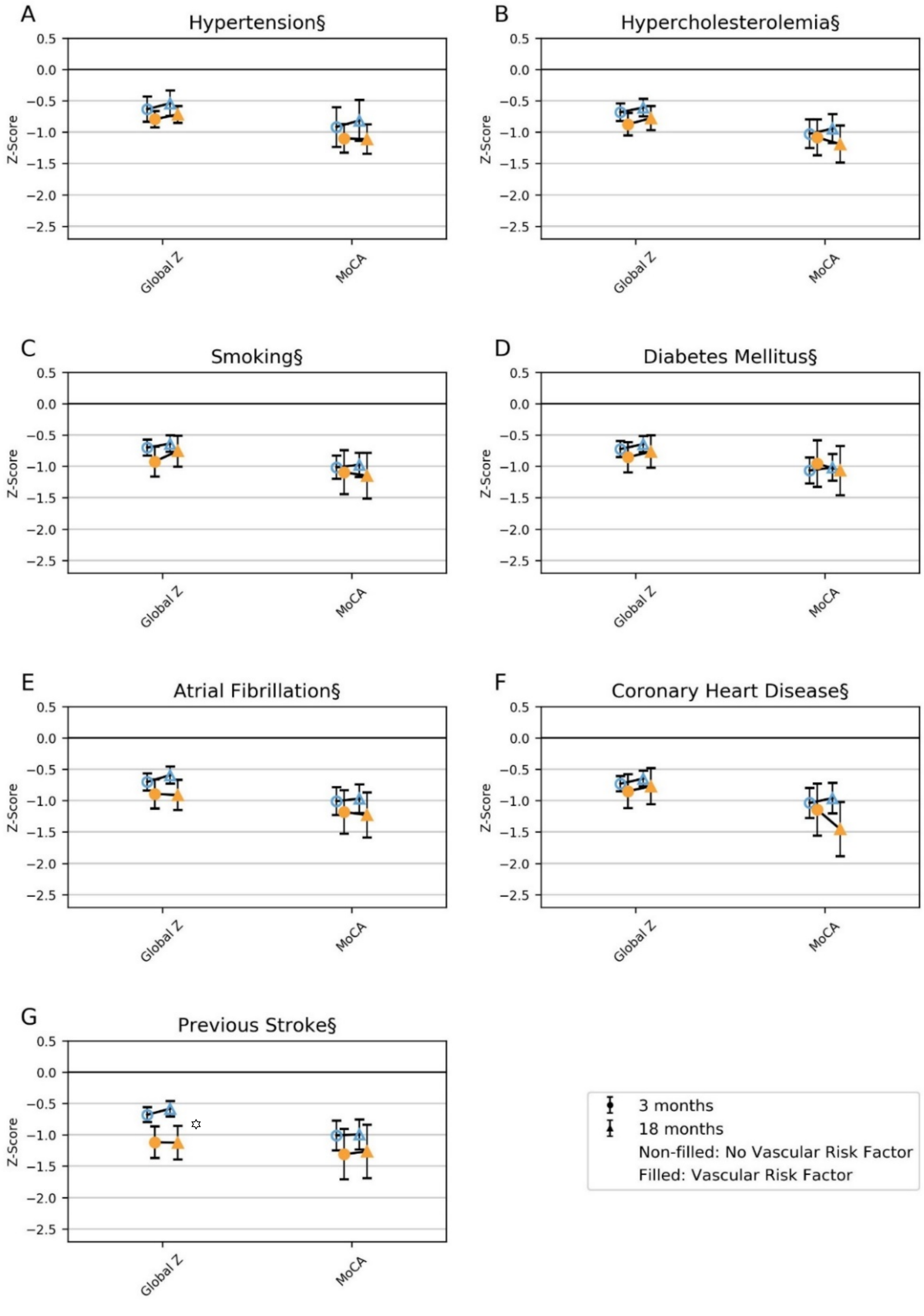


**Figure S6. Sensitivity analyses with exclusion of patients with pre-stroke dementia: Mean z-scores with 95% confidence intervals for the global cognitive measures for the different vascular risk factors at 3- and 18-months post-stroke, adjusted for age, education and sex in model 1**

MoCA = Montreal Cognitive Assessment

§ exclusion of patients with pre-stroke dementia, defined as pre-stroke Global Deterioration Scale 4-7, adjusted for age, education, and sex

✡LRvasc $\chi$2(2) p<0.01, ✡✡LRtime $\chi$2(2) p<0.01

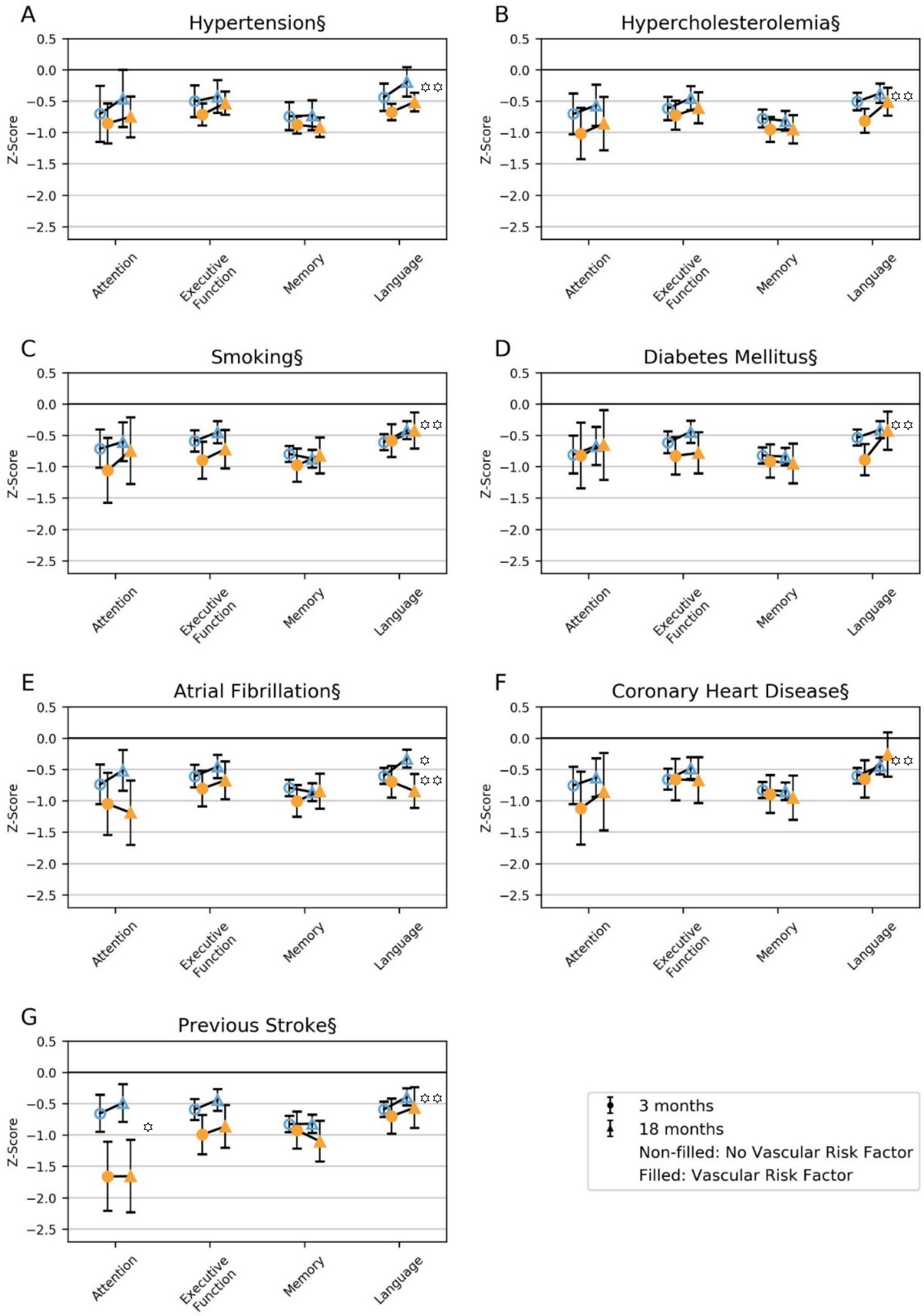


**Figure S7. Sensitivity analyses with exclusion of patients with pre-stroke dementia: Mean z-scores with 95% confidence intervals for the cognitive domains for the different vascular risk factors at 3- and 18-months post-stroke, adjusted for age, education and sex in model 1**

§ exclusion of patients with pre-stroke dementia, defined as pre-stroke Global Deterioration Scale 4-7, adjusted for age, education, and sex

✡LRvasc $\chi2(2)$ p<0.01, ✡✡LRtime $\chi2(2)$ p<0.01

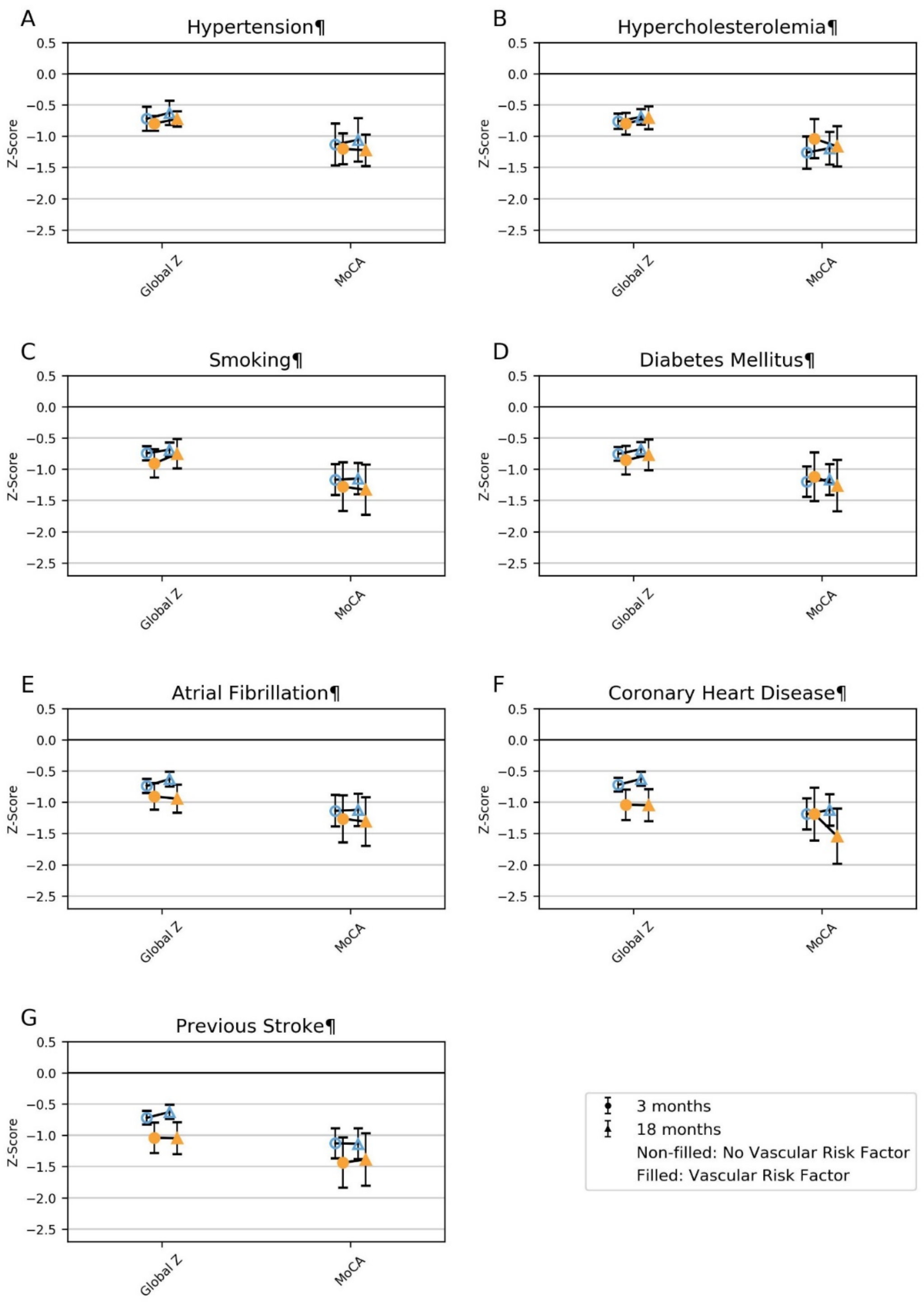


**Figure S8. Sensitivity analyses with adjustment for age, education, sex, pre-stroke mRS, and NIHSS: Mean z-scores with 95% confidence intervals for the global cognitive measures for the different vascular risk factors at 3- and 18-months post-stroke in model 1**

MoCA = Montreal Cognitive Assessment

¶ adjusted for age, education and sex, pre-stroke modified Rankin Scale (mRS), and National Institutes of Health Stroke Scale (NIHSS)

☆LRvasc χ2(2) p<0.01, ☆☆LRtime χ2(2) p<0.01

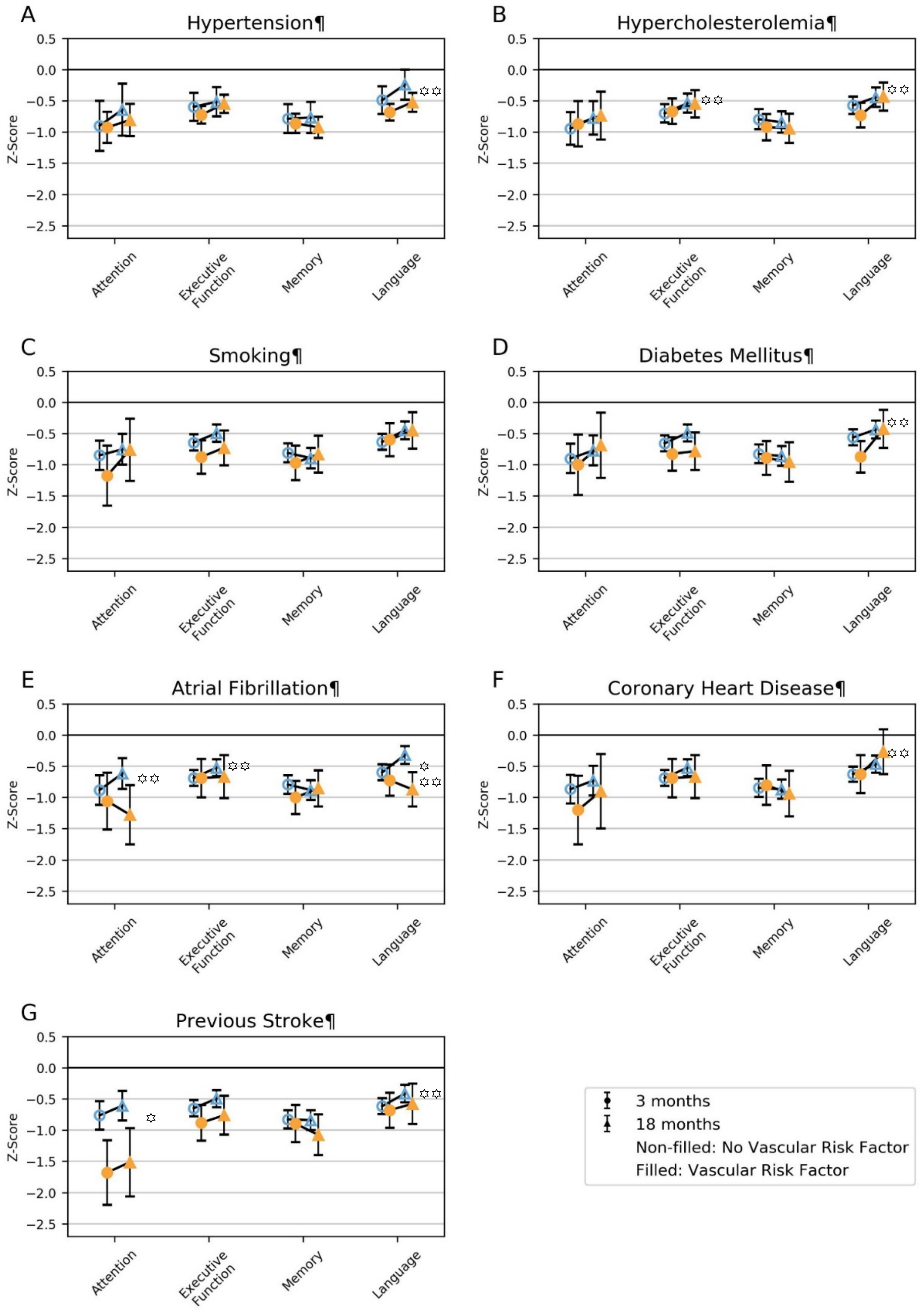


**Figure S9. Sensitivity analyses with adjustment for age, education, sex, pre-stroke mRS, and NIHSS: Mean z-scores with 95% confidence intervals for the cognitive domains for the different vascular risk factors at 3- and 18-months post-stroke in model 1**

¶ adjusted for age, education and sex, pre-stroke modified Rankin Scale (mRS), and, National Institutes of Health Stroke Scale (NIHSS)

☼LRvasc $\chi2(2)$ p<0.01, ☼☼LRtime $\chi2(2)$ p<0.01

| Table S3. Hypothesis test of whether there is an effect of the vascular risk factor and follow-up time in model 1 for the global measures and cognitive domains for the different vascular risk factors for unadjusted analyses | | | | | | | | |
|---|---|---|---|---|---|---|---|---|
| | | **Hypertension** | **Hypercholesterolemia** | **Smoking** | **Diabetes mellitus** | **Atrial fibrillation** | **Coronary heart disease** | **Previous stroke** |
| **Global z** | $LR_{vasc}$ χ2(2), p-value | 4.89, 0.087 | 6.22, 0.045 | 3.02, 0.221 | 3.60, 0.165 | 10.68, 0.005* | 4.95, 0.084 | 17.40, <0.001* |
| | $LR_{time}$ χ2(2), p-value | 6.29, 0.043 | 5.97, 0.050 | 7.64, 0.022 | 6.00, 0.050 | 9.07, 0.011 | 6.08, 0.048 | 7.21, 0.027 |
| **MoCA** | $LR_{vasc}$ χ2(2), p-value | 8.60, 0.014 | 5.73, 0.057 | 0.11, 0.948 | 3.84, 0.147 | 10.54, 0.005* | 20.77, <0.001* | 10,89, 0.004 |
| | $LR_{time}$ χ2(2), p-value | 0.73., 0.695 | 2.31, 0.315 | 0.26, 0.880 | 1.46, 0.481 | 0.55, 0.758 | 6.64, 0.036 | 0.27, 0.872 |
| **Attention** | $LR_{vasc}$ χ2(2), p-value | 4.44, 0.109 | 3.89, 0.143 | 3.73, 0.155 | 3.14, 0.209 | 12.61, 0.002* | 7.92, 0.019 | 18.07, <0.001* |
| | $LR_{time}$ χ2(2), p-value | 6.03, 0.049 | 5.38, 0.068 | 8.45, 0.015 | 6.19, 0.045 | 11.39, 0.003* | 5.96, 0.051 | 5.25, 0.073 |
| **Executive function** | $LR_{vasc}$ χ2(2), p-value | 2.48, 0.290 | 1.45, 0.484 | 5.09, 0.079 | 4.66, 0.097 | 2.26, 0.323 | 1.87, 0.393 | 8.19, 0.017 |
| | $LR_{time}$ χ2(2), p-value | 8.96, 0.011 | 8.32, 0.016 | 8.14, 0.017 | 8.80, 0.012 | 8.31, 0.016 | 9.50, 0.009* | 8.20, 0.017 |
| **Memory** | $LR_{vasc}$ χ2(2), p-value | 3.90, 0.142 | 4.78, 0.093 | 2.44, 0.295 | 1.72, 0.424 | 4.92, 0.027 | 1.88, 0.170 | 3.54, 0.060 |

| | | | | | | | | |
|---|---|---|---|---|---|---|---|---|
| | $LR_{time}$ χ2(2), p-value | 0.41, 0.937 | 0.12, 0.944 | 2.32, 0.128 | 0.04, 0.998 | 2.16, 0.142 | 0.15, 0.696 | 1.07, 0.585 |
| **Language** | $LR_{vasc}$ χ2(2), p-value | 9.35, 0.009* | 7.26, 0.027 | 0.21, 0.901 | 9.32, 0.001* | 16.14, <0.001* | 1.96, 0.376 | 2.83, 0.243 |
| | $LR_{time}$ χ2(2), p-value | 10.41, 0.006* | 11.68, 0.003* | 9.94, 0.007* | 13.96, <0.001* | 18.97, <0.001* | 11.59, 0.003* | 10.35, 0.006* |

MoCA=Montreal Cognitive Assessment.
$LR_{vasc}$ $\chi2(2)$ =Likelihood ratio test model 1 vs model 2, with two degrees of freedom; hypothesis test of whether there is an effect of the vascular risk factor.
$LR_{time}$ $\chi2(2)$ =Likelihood ratio test model 1 vs model 3, with two degrees of freedom; hypothesis test of whether there is an effect of follow-up time.
*p<0.01

| Table S4. Hypothesis test of whether there is an effect of the vascular risk factor and follow-up time in model 1 for the global measures and cognitive domains for the different vascular risk factors for analyses with exclusion of deceased at 18 months adjusted for age, education and sex | | | | | | | | |
|---|---|---|---|---|---|---|---|---|
| | | **Hypertension** | **Hypercholesterolemia** | **Smoking** | **Diabetes mellitus** | **Atrial fibrillation** | **Coronary heart disease** | **Previous stroke** |
| **Global z** | $LR_{vasc}$ $\chi2(2)$, p-value | 1.56, 0.458 | 2.39, 0.302 | 4.32, 0.116 | 1.42, 0.491 | 6.79, 0.034 | 1.09, 0.579 | 12.76, 0.002* |
| | $LR_{time}$ $\chi2(2)$, p-value | 5.27, 0.072 | 5.30, 0.071 | 6.65, 0.036 | 5.02, 0.081 | 7.95, 0.019 | 5.16, 0.076 | 6.40, 0.041 |
| **MoCA** | $LR_{vasc}$ $\chi2(2)$, p-value | 1.63, 0.653 | 1.94, 0.380 | 1.41, 0.236 | 1.64, 0.651 | 1.62, 0.655 | 8.50, 0.014 | 6.08, 0.108 |
| | $LR_{time}$ $\chi2(2)$, p-value | 0.63, 0.730 | 1.90, 0.386 | 0.13, 0.723 | 1.00, 0.608 | 0.35, 0.840 | 6.69, <0.01* | 0.13, 0.936 |
| **Attention** | $LR_{vasc}$ $\chi2(2)$, p-value | 1.21, 0.546 | 0.75, 0.687 | 4.24, 0.120 | 1.71, 0.425 | 5.59, 0.014 | 1.53, 0.466 | 14.09, 0.001* |
| | $LR_{time}$ $\chi2(2)$, p-value | 5.09, 0.079 | 4.51, 0.105 | 7.36, 0.025 | 5.33, 0.070 | 10.36, <0.001* | 4.16, 0.125 | 4.31, 0.116 |
| **Executive function** | $LR_{vasc}$ $\chi2(2)$, p-value | 2.59, 0.274 | 1.04, 0.595 | 4.93, 0.085 | 4.19, 0.123 | 2.55, 0.279 | 1.64, 0.442 | 7.20, 0.027 |
| | $LR_{time}$ $\chi2(2)$, p-value | 8.51, 0.014 | 7.87, 0.020 | 7.55, 0.023 | 8.27, 0.016 | 7.79, 0.020 | 8.98, 0.011 | 7.69, 0.021 |

| | | | | | | | | |
|---|---|---|---|---|---|---|---|---|
| **Memory** | $LR_{vasc}$ $\chi2(2)$, p-value | 1.63, 0.442 | 0.71, 0.400 | 3.33, 0.189 | 0.28, 0.870 | 3.58, 0.167 | 0.40, 0.528 | 2.13, 0.144 |
| | $LR_{time}$ $\chi2(2)$, p-value | 0.46, 0.796 | 0.18, 0.912 | 2.87, 0.090 | 0.28, 0.964 | 2.51, 0.285 | 0.35, 0.839 | 1.76, 0.184 |
| **Language** | $LR_{vasc}$ $\chi2(2)$, p-value | 5.42, 0.067 | 3.41, 0.182 | 0.21, 0.900 | 7.12, 0.028 | 12.65, 0.002* | 1.51, 0.470 | 1.43, 0.489 |
| | $LR_{time}$ $\chi2(2)$, p-value | 9.50, 0.009* | 10.48, 0.005* | 8.83, 0.012 | 12.79, 0.002* | 18.05, <0.001* | 10.63, 0.005* | 9.47, 0.009* |

MoCA=Montreal Cognitive Assessment.
$LR_{vasc}$ $\chi2(2)$ =Likelihood ratio test model 1 vs model 2, with two degrees of freedom; hypothesis test of whether there is an effect of the vascular risk factor.
$LR_{time}$ $\chi2(2)$ =Likelihood ratio test model 1 vs model 3, with two degrees of freedom; hypothesis test of whether there is an effect of follow-up time.
*p<0.01

| **Table S5. Hypothesis test of whether there is an effect of the vascular risk factor and time in model 1 for the global measures and cognitive domains for the different vascular risk factors for analyses with exclusion of pre-stroke dementia adjusted for age, education and sex** | | | | | | | | |
|---|---|---|---|---|---|---|---|---|
| | | **Hypertension** | **Hypercholesterolemia** | **Smoking** | **Diabetes mellitus** | **Atrial fibrillation** | **Coronary heart disease** | **Previous stroke** |
| **Global z** | $LR_{vasc}$ χ2(2), p-value | 2.30, 0.317 | 3.07, 0.215 | 3.41, 0.182 | 0.98, 0.612 | 6.02, 0.050 | 0.71, 0.701 | 13.93, <0.001* |
| | $LR_{time}$ χ2(2), p-value | 5.56, 0.062 | 5.53, 0.063 | 7.23, 0.027 | 5.47, 0.065 | 7.74, 0.021 | 5.48, 0.065 | 6.61, 0.037 |
| **MoCA** | $LR_{vasc}$ χ2(2), p-value | 2.87, 0.239 | 3.00, 0.223 | 0.80, 0.670 | 1.03, 0.596 | 1.82, 0.402 | 6.92, 0.031 | 2.27, 0.321 |
| | $LR_{time}$ χ2(2), p-value | 0.83, 0.662 | 2.25, 0.325 | 0.51, 0.776 | 1.08, 0.582 | 0.46, 0.793 | 5.27, 0.072 | 0.12, 0.941 |
| **Attention** | $LR_{vasc}$ χ2(2), p-value | 1.64, 0.440 | 1.98, 0.372 | 2.17, 0.338 | 0.02, 0.988 | 7.54, 0.02 | 2.12, 0.347 | 15.70, <0.001* |
| | $LR_{time}$ χ2(2), p-value | 4.62, 0.099 | 3.79, 0.150 | 5.33, 0.070 | 3.87, 0.145 | 8.11, 0.017 | 5.11, 0.078 | 4.37, 0.113 |
| **Executive function** | $LR_{vasc}$ χ2(2), p-value | 2.45, 0.294 | 1.24, 0.539 | 3.96, 0.138 | 3.80, 0.150 | 2.15, 0.342 | 1.57, 0.453 | 7.12, 0.028 |
| | $LR_{time}$ χ2(2), p-value | 8.50, 0.014 | 7.78, 0.020 | 7.59, 0.023 | 8.26, 0.016 | 7.79, 0.020 | 9.12, 0.011 | 7.65, 0.022 |

| | | | | | | | | |
|---|---|---|---|---|---|---|---|---|
| **Memory** | $LR_{vasc}$ $\chi 2(2)$, p-value | 1.91, 0.592 | 1.99, 0.575 | 2.68, 0.261 | 0.50, 0.918 | 3.15, 0.369 | 0.29, 0.962 | 2.44, 0.486 |
| | $LR_{time}$ $\chi 2(2)$, p-value | † | 0.22, 0.895 | 2.56, 0.278 | 0.15, 0.929 | 2.42, 0.490 | 0.17, 0.917 | 1.26, 0.532 |
| **Language** | $LR_{vasc}$ $\chi 2(2)$, p-value | 5.56, 0.062 | 6.48, 0.039 | 0.04, 0.980 | 7.71, 0.021 | 12.89, 0.002* | 1.77, 0.412 | 0.94, 0.0624 |
| | $LR_{time}$ $\chi 2(2)$, p-value | 9.86, 0.007* | 11.55, 0.003* | 9.26, <0.001* | 13.75, 0.001* | 19.43, <0.001* | 11.43, 0.003* | 9.71, 0.008* |

MoCA=Montreal Cognitive Assessment.
$LR_{vasc}$ $\chi 2(2)$ =Likelihood ratio test model 1 vs model 2, with two degrees of freedom; hypothesis test of whether there is an effect of the vascular risk factor.
$LR_{time}$ $\chi 2(2)$ =Likelihood ratio test model 1 vs model 3, with two degrees of freedom; hypothesis test of whether there is an effect of follow-up time.
*p<0.01
† calculations did not converge

| **Table S6. Hypothesis test of whether there is an effect of the vascular risk factor and follow-up time in model 1 for the global measures and cognitive domains for the different vascular risk factors for analyses adjusted for age, education, sex, pre-stroke mRS and NIHSS** | | | | | | | | |
|---|---|---|---|---|---|---|---|---|
| | | **Hypertension** | **Hypercholesterolemia** | **Smoking** | **Diabetes mellitus** | **Atrial fibrillation** | **Coronary heart disease** | **Previous stroke** |
| **Global z** | $LR_{vasc}$ χ2(2), p-value | 0.71, 0.703 | 0.18, 0.913 | 2.15, 0.341 | 0.61, 0.738 | 6.45, 0.040 | 0.35, 0.841 | 8.50, 0.014 |
| | $LR_{time}$ χ2(2), p-value | 5.09, 0.079 | 5.07, 0.079 | 6.14, 0.046 | 4.94, 0.085 | 7.89, 0.019 | 5.17, 0.076 | 6.10, 0.047 |
| **MoCA** | $LR_{vasc}$ χ2(2), p-value | 0.99, 0.608 | 3.06, 0.217 | 0.77, 0.682 | 0.99, 0.608 | 0.89, 0.642 | 6.92, 0.031 | 2.30, 0.317 |
| | $LR_{time}$ χ2(2), p-value | 0.52, 0.771 | 2.25, 0.324 | 0.21, 0.901 | 0.52, 0.771 | 0.16, 0.923 | 6.17, 0.046 | 0.12, 0.942 |
| **Attention** | $LR_{vasc}$ χ2(2), p-value | 0.75, 0.686 | 0.13, 0.939 | 3.16, 0.207 | 0.82, 0.664 | 8.82, 0.012 | 1.37, 0.503 | 10.78, 0.005* |
| | $LR_{time}$ χ2(2), p-value | 4.70, 0.095 | 4.15, 0.125 | 6.76, 0.034 | 4.88, 0.087 | 10.73, 0.005* | 4.43, 0.104 | 4.04, 0.133 |
| **Executive function** | $LR_{vasc}$ χ2(2), p-value | 1.14, 0.767 | 0.17, 0.916 | 2.67, 0.263 | 3.04, 0.386 | 1.41, 0.494 | 0.93, 0.819 | 2.73, 0.435 |
| | $LR_{time}$ χ2(2), p-value | 8.18, 0.043 | 7.72, <0.001* | 7.54, 0.023 | 8.10, 0.018 | 7.55, 0.006* | 8.32, 0.016 | 7.55, 0.056 |

| | | | | | | | | |
|---|---|---|---|---|---|---|---|---|
| **Memory** | $LR_{vasc}$ $\chi2(2)$, p-value | 1.17, 0.0558 | 1.01, 0.603 | 2.38, 0.304 | 0.33, 0.847 | 3.24, 0.176 | 0.36, 0.834 | 1.88, 0.390 |
| | $LR_{time}$ $\chi2(2)$, p-value | 0.77, 0.680 | 0.46, 0.795 | 2.52, 0.283 | 0.45, 0.799 | 2.90, 0.234 | 0.75, 0.687 | 1.42, 0.491 |
| **Language** | $LR_{vasc}$ $\chi2(2)$, p-value | 3.93, 0.139 | 2.28, 0.319 | 0.10, 0.951 | 6.31, 0.043 | 13.32, 0.001* | 1.34, 0.511 | 0.83, 0.660 |
| | $LR_{time}$ $\chi2(2)$, p-value | 9.63, 0.008* | 10.88, 0.004* | 8.90, 0.012 | 13.06, 0.002* | 18.03, <0.001* | 10.54, <0.01* | 9.66, 0.008* |

MoCA=Montreal Cognitive Assessment.

$LR_{vasc}$ $\chi2(2)$ =Likelihood ratio test model 1 vs model 2, with two degrees of freedom; hypothesis test of whether there is an effect of the vascular risk factor.

$LR_{time}$ $\chi2(2)$ =Likelihood ratio test model 1 vs model 3, with two degrees of freedom; hypothesis test of whether there is an effect of follow-up time.

*p<0.01

**Table S7. Numbers of patients with the different vascular risk factors included in the analyses**

| | | | HTN | | | HC | | | S | | | DM | | | AF | | | CHD | | | PS | | |
|---|---|---|---|---|---|---|---|---|---|---|---|---|---|---|---|---|---|---|---|---|---|---|---|
| | | | N | Y | T | N | Y | T | N | Y | T | N | Y | T | N | Y | T | N | Y | T | N | Y | T |
| **Global z** | Unadjusted and adjusted for age, education, and sex | 3 months | 156 | 409 | 565 | 365 | 200 | 565 | 450 | 112 | 562 | 457 | 108 | 565 | 438 | 127 | 565 | 474 | 91 | 565 | 468 | 97 | 565 |
| | | 18 months | 132 | 320 | 452 | 308 | 144 | 452 | 356 | 93 | 449 | 380 | 72 | 452 | 355 | 97 | 452 | 392 | 60 | 452 | 382 | 70 | 452 |
| | Exclusion of deceased (n=17) adjusted for age, education, and sex | 3 months | 152 | 396 | 548 | 357 | 191 | 548 | 435 | 110 | 545 | 445 | 103 | 548 | 461 | 87 | 548 | 461 | 87 | 548 | 455 | 93 | 548 |
| | | 18 months | 132 | 320 | 452 | 308 | 144 | 452 | 356 | 93 | 449 | 380 | 72 | 452 | 392 | 60 | 452 | 392 | 60 | 452 | 382 | 70 | 452 |
| | Exclusion of pre-stroke dementia (n=12) adjusted for age, education, and sex | 3 months | 153 | 400 | 553 | 358 | 195 | 553 | 441 | 109 | 550 | 449 | 104 | 553 | 431 | 122 | 553 | 466 | 87 | 553 | 461 | 92 | 553 |
| | | 18 months | 132 | 315 | 447 | 305 | 142 | 447 | 353 | 91 | 444 | 376 | 71 | 447 | 352 | 95 | 447 | 389 | 58 | 447 | 380 | 67 | 447 |
| | Adjusted for age, education, sex, pre-stroke mRS, and NIHSS | 3 months | 152 | 397 | 549 | 355 | 194 | 549 | 440 | 108 | 548 | 443 | 106 | 549 | 427 | 122 | 549 | 463 | 86 | 549 | 453 | 96 | 549 |
| | | 18 months | 130 | 314 | 444 | 303 | 141 | 444 | 351 | 92 | 443 | 372 | 72 | 444 | 350 | 94 | 444 | 387 | 57 | 444 | 374 | 70 | 444 |
| **MoCA** | Unadjusted and adjusted for age, education, and sex | 3 months | 168 | 437 | 605 | 393 | 209 | 605 | 486 | 116 | 602 | 491 | 114 | 605 | 465 | 140 | 605 | 501 | 104 | 605 | 496 | 109 | 605 |
| | | 18 months | 141 | 367 | 508 | 339 | 169 | 508 | 406 | 99 | 505 | 422 | 86 | 508 | 395 | 113 | 508 | 428 | 80 | 508 | 427 | 81 | 508 |
| | Exclusion of deceased (n=20) adjusted for age, education, and sex | 3 months | 162 | 423 | 585 | 385 | 200 | 585 | 469 | 113 | 582 | 478 | 105 | 583 | 485 | 100 | 585 | 486 | 97 | 583 | 483 | 102 | 585 |
| | | 18 months | 141 | 367 | 508 | 339 | 169 | 369 | 406 | 99 | 505 | 415 | 81 | 496 | 428 | 80 | 508 | 421 | 75 | 496 | 427 | 81 | 508 |
| | Exclusion of pre-stroke dementia (n=22) adjusted for age, education, and sex | 3 months | 163 | 420 | 583 | 381 | 202 | 583 | 468 | 112 | 580 | 478 | 105 | 583 | 451 | 132 | 583 | 486 | 97 | 583 | 484 | 99 | 583 |
| | | 18 months | 139 | 367 | 496 | 331 | 165 | 496 | 397 | 96 | 493 | 415 | 81 | 496 | 388 | 108 | 496 | 421 | 75 | 496 | 421 | 75 | 496 |

| | | | | | | | | | | | | | | | | | | | | | | | |
|---|---|---|---|---|---|---|---|---|---|---|---|---|---|---|---|---|---|---|---|---|---|---|---|
| | Adjusted for age, education, sex, pre-stroke mRS, and NIHSS | 3 months | 164 | 424 | 588 | 385 | 203 | 588 | 475 | 112 | 587 | 477 | 111 | 588 | 454 | 134 | 588 | 489 | 99 | 588 | 480 | 108 | 588 |
| | | 18 months | 138 | 659 | 497 | 331 | 166 | 497 | 398 | 98 | 496 | 412 | 85 | 497 | 388 | 109 | 497 | 420 | 77 | 497 | 416 | 81 | 497 |
| **Attention** | Unadjusted and adjusted for age, education, and sex | 3 months | 155 | 410 | 565 | 367 | 198 | 565 | 452 | 110 | 562 | 155 | 410 | 565 | 437 | 128 | 565 | 473 | 92 | 565 | 469 | 96 | 565 |
| | | 18 months | 131 | 323 | 454 | 309 | 145 | 454 | 360 | 91 | 451 | 131 | 323 | 454 | 354 | 100 | 454 | 394 | 60 | 454 | 385 | 69 | 454 |
| | Exclusion of deceased (n=18) adjusted for age, education, and sex | 3 months | 150 | 397 | 547 | 358 | 189 | 547 | 436 | 108 | 544 | 150 | 397 | 547 | 425 | 122 | 547 | 459 | 88 | 547 | 456 | 91 | 547 |
| | | 18 months | 131 | 323 | 454 | 309 | 145 | 454 | 360 | 91 | 451 | 131 | 323 | 454 | 354 | 100 | 454 | 394 | 60 | 454 | 385 | 69 | 454 |
| | Exclusion of pre-stroke dementia (n=14) adjusted for age, education, and sex | 3 months | 153 | 398 | 551 | 358 | 193 | 551 | 442 | 106 | 548 | 153 | 398 | 551 | 428 | 123 | 551 | 464 | 87 | 551 | 461 | 90 | 551 |
| | | 18 months | 131 | 319 | 450 | 307 | 143 | 450 | 358 | 89 | 447 | 131 | 319 | 450 | 352 | 98 | 450 | 392 | 58 | 450 | 383 | 67 | 450 |
| | Adjusted for age, education, sex, pre-stroke mRS, and NIHSS | 3 months | 151 | 399 | 550 | 357 | 193 | 550 | 441 | 107 | 548 | 151 | 399 | 550 | 428 | 122 | 550 | 463 | 87 | 550 | 455 | 95 | 550 |
| | | 18 months | 129 | 317 | 446 | 304 | 142 | 446 | 355 | 90 | 445 | 129 | 317 | 446 | 349 | 97 | 446 | 389 | 57 | 446 | 377 | 69 | 446 |
| **Executive function** | Unadjusted and adjusted for age, education, and sex | 3 months | 151 | 407 | 558 | 358 | 200 | 558 | 445 | 110 | 555 | 450 | 108 | 558 | 432 | 126 | 558 | 468 | 90 | 558 | 450 | 108 | 558 |
| | | 18 months | 132 | 318 | 450 | 308 | 142 | 450 | 355 | 92 | 447 | 378 | 72 | 450 | 353 | 97 | 450 | 391 | 59 | 450 | 378 | 72 | 450 |
| | Exclusion of deceased (n=17) adjusted for age, education, and sex | 3 months | 147 | 394 | 541 | 350 | 191 | 541 | 430 | 108 | 538 | 147 | 394 | 541 | 420 | 121 | 541 | 455 | 86 | 541 | 449 | 92 | 541 |
| | | 18 months | 132 | 318 | 450 | 308 | 142 | 450 | 355 | 92 | 447 | 132 | 318 | 450 | 353 | 97 | 450 | 391 | 59 | 450 | 380 | 70 | 450 |
| | Exclusion of pre-stroke dementia (n=11) adjusted for age, education, and sex | 3 months | 149 | 398 | 547 | 352 | 195 | 547 | 436 | 106 | 542 | 443 | 104 | 547 | 426 | 121 | 547 | 461 | 86 | 547 | 456 | 91 | 547 |
| | | 18 months | 132 | 313 | 445 | 305 | 140 | 445 | 350 | 91 | 441 | 374 | 71 | 445 | 350 | 95 | 445 | 388 | 57 | 445 | 378 | 67 | 445 |

| | | | | | | | | | | | | | | | | | | | | | | | |
|---|---|---|---|---|---|---|---|---|---|---|---|---|---|---|---|---|---|---|---|---|---|---|---|
| | Adjusted for age, education, sex, pre-stroke mRS, and NIHSS | 3 months | 148 | 395 | 543 | 349 | 194 | 543 | 436 | 106 | 542 | 437 | 106 | 543 | 422 | 121 | 543 | 458 | 85 | 543 | 437 | 106 | 543 |
| | | 18 months | 130 | 312 | 442 | 303 | 139 | 442 | 350 | 91 | 441 | 370 | 72 | 442 | 348 | 94 | 442 | 386 | 56 | 442 | 370 | 72 | 442 |
| **Memory** | Unadjusted and adjusted for age, education, and sex | 3 months | 137 | 355 | 492 | 318 | 174 | 492 | 393 | 97 | 490 | 395 | 97 | 492 | 383 | 109 | 492 | 415 | 77 | 492 | 412 | 80 | 492 |
| | | 18 months | 110 | 255 | 365 | 251 | 114 | 365 | 287 | 75 | 362 | 307 | 58 | 365 | 288 | 77 | 365 | 317 | 48 | 365 | 306 | 59 | 365 |
| | Exclusion of deceased (n=15) adjusted for age, education, and sex | 3 months | 133 | 344 | 477 | 311 | 166 | 477 | 380 | 95 | 475 | 384 | 93 | 477 | 373 | 104 | 477 | 403 | 74 | 477 | 401 | 76 | 477 |
| | | 18 months | 110 | 255 | 365 | 251 | 114 | 365 | 287 | 75 | 362 | 307 | 58 | 365 | 288 | 77 | 365 | 317 | 48 | 365 | 306 | 59 | 365 |
| | Exclusion of pre-stroke dementia (n=10) adjusted for age, education, and sex | 3 months | 134 | 348 | 482 | 312 | 170 | 482 | 386 | 94 | 480 | 387 | 95 | 482 | 377 | 105 | 482 | 407 | 75 | 482 | 407 | 75 | 482 |
| | | 18 months | 110 | 251 | 361 | 248 | 113 | 361 | 284 | 74 | 358 | 305 | 56 | 361 | 285 | 76 | 361 | 315 | 46 | 361 | 305 | 56 | 361 |
| | Adjusted for age, education, sex, pre-stroke mRS, and NIHSS | 3 months | 133 | 346 | 479 | 310 | 169 | 479 | 383 | 95 | 478 | 384 | 95 | 479 | 375 | 104 | 479 | 407 | 72 | 479 | 400 | 79 | 479 |
| | | 18 months | 108 | 249 | 357 | 246 | 111 | 357 | 282 | 74 | 356 | 299 | 58 | 357 | 283 | 74 | 357 | 312 | 45 | 357 | 298 | 59 | 357 |
| **Language** | Unadjusted and adjusted for age, education, and sex | 3 months | 130 | 350 | 480 | 310 | 170 | 480 | 383 | 94 | 477 | 381 | 99 | 480 | 379 | 101 | 480 | 407 | 73 | 480 | 398 | 82 | 480 |
| | | 18 months | 101 | 238 | 339 | 230 | 109 | 339 | 267 | 69 | 336 | 284 | 55 | 339 | 262 | 77 | 339 | 294 | 45 | 339 | 287 | 52 | 339 |
| | Exclusion of deceased (n=15) adjusted for age, education, and sex | 3 months | 126 | 339 | 465 | 303 | 162 | 465 | 370 | 92 | 462 | 370 | 95 | 465 | 369 | 96 | 465 | 395 | 70 | 465 | 387 | 78 | 465 |
| | | 18 months | 101 | 238 | 339 | 230 | 109 | 339 | 267 | 69 | 336 | 284 | 55 | 339 | 262 | 77 | 339 | 294 | 45 | 339 | 287 | 52 | 339 |
| | Exclusion of pre-stroke dementia (n=9) adjusted for age, education, and sex | 3 months | 129 | 342 | 471 | 306 | 165 | 471 | 376 | 92 | 468 | 374 | 97 | 471 | 374 | 97 | 471 | 401 | 70 | 471 | 394 | 77 | 471 |
| | | 18 months | 101 | 234 | 355 | 228 | 107 | 335 | 265 | 67 | 332 | 281 | 54 | 335 | 259 | 76 | 335 | 292 | 43 | 335 | 286 | 49 | 335 |

| | | | | | | | | | | | | | | | | | | | | | | | |
|---|---|---|---|---|---|---|---|---|---|---|---|---|---|---|---|---|---|---|---|---|---|---|---|
| | Adjusted for age, education, sex, pre-stroke mRS, and NIHSS | 3 months | 127 | 340 | 467 | 302 | 165 | 467 | 376 | 92 | 468 | 370 | 97 | 467 | 369 | 98 | 467 | 399 | 68 | 467 | 385 | 82 | 467 |
| | | 18 months | 99 | 232 | 331 | 225 | 106 | 331 | 265 | 67 | 332 | 276 | 55 | 331 | 262 | 77 | 339 | 289 | 42 | 331 | 279 | 52 | 331 |

HTN = Hypertension, HC = Hypercholesterolemia, S = Smoking, DM = Diabetes mellitus, AF = Atrial fibrillation, CHD = Coronary heart disease, PS = Previous stroke, N = No, Y = Yes, T = Total, MoCA= Montreal Cognitive Assessment, mRS = modified Rankin Scale, NIHSS = National Institutes of Health Stroke Scale

## References


1. Munthe-Kaas R, Aam S, Ihle-Hansen H, Lydersen S, Knapskog A-B, Wyller TB, et al. Impact of different methods defining post-stroke neurocognitive disorder: The Nor-COAST study. Alzheimer's Dement. 2020;6(1):e12000.
2. Aam S, Einstad MS, Munthe-Kaas R, Lydersen S, Ihle-Hansen H, Knapskog AB, et al. Post-stroke cognitive impairment: Impact of follow-up time and stroke subtype on severity and cognitive profile: The Nor-COAST Study. Front Neurol. 2020;11:699.
3. Pendlebury ST, Welch SJV, Cuthbertson FC, Mariz J, Mehta Z, Rothwell PM. Telephone assessment of cognition after transient ischemic attack and stroke: Modified telephone interview of cognitive status and telephone Montreal Cognitive Assessment versus face-to-face Montreal Cognitive Assessment and Neuropsychological Battery. Stroke. 2013;44(1):227-9.
4. Teuschl Y, Ihle-Hansen H, Matz K, Dachenhausen A, Ratajczak P, Tuomilehto J, et al. Multidomain intervention for the prevention of cognitive decline after stroke – A pooled patient-level data analysis. Eur J Neurol. 2018;25(9):1182-8.
5. Pendlebury ST, Chen PJ, Welch SJ, Cuthbertson FC, Wharton RM, Mehta Z, et al. Methodological Factors in Determining Risk of Dementia After Transient Ischemic Attack and Stroke: (II) Effect of Attrition on Follow-Up. Stroke. 2015;46(6):1494-500.
6. Veierød MB, Lydersen S, Laake P. Medical statistics in clinical and epidemiological research: Gyldendal akademisk; 2012.
7. Tombaugh TN. Trail Making Test A and B: Normative data stratified by age and education. Arch Clin Neuropsychol. 2004;19(2):203-14.
8. Luck T, Pabst A, Rodriguez FS, Schroeter ML, Witte V, Hinz A, et al. Age-, sex-, and education-specific norms for an extended CERAD Neuropsychological Assessment Battery-Results from the population-based LIFE-Adult-Study. Neuropsychology. 2018;32(4):461-75.
9. Welsh KA, Butters N, Mohs RC, Beekly D, Edland S, Fillenbaum G, et al. The Consortium to Establish a Registry for Alzheimer's Disease (CERAD). Part V. A normative study of the neuropsychological battery. Neurology. 1994;44(4):609-14.
10. Tombaugh TN, Kozak J, Rees L. Normative data stratified by age and education for two measures of verbal fluency: FAS and animal naming. Arch Clin Neuropsychol. 1999;14(2):167-77.
11. Borland E, Nagga K, Nilsson PM, Minthon L, Nilsson ED, Palmqvist S. The Montreal Cognitive Assessment: Normative Data from a Large Swedish Population-Based Cohort. J Alzheimers Dis. 2017;59(3):893-901.
